# MBZM-N-IBT, a Novel Small Molecule, Restricts Chikungunya Virus Infection by Targeting nsP2 Protease Activity *In Vitro*, *In Vivo*, and *Ex Vivo*

**DOI:** 10.1128/aac.00463-22

**Published:** 2022-06-29

**Authors:** Saikat De, Soumyajit Ghosh, Supriya Suman Keshry, Chandan Mahish, Chinmayee Mohapatra, Ankeeta Guru, Prabhudutta Mamidi, Ankita Datey, Sweta Smita Pani, Dileep Vasudevan, Tushar Kant Beuria, Subhasis Chattopadhyay, Bharat Bhusan Subudhi, Soma Chattopadhyay

**Affiliations:** a Institute of Life Sciences, Bhubaneswar, Odisha, India; b Regional Centre for Biotechnology, Faridabad, Haryana, India; c School of Biotechnology, Kalinga Institute of Industrial Technology (KIIT) University, Bhubaneswar, Odisha, India; d National Institute of Science Education and Research, Bhubaneswar, Odisha, India; e Homi Bhabha National Institute, Training School Complex, Mumbai, Maharashtra, India; f School of Pharmaceutical Sciences, Siksha O Anusandhan Deemed to be University, Bhubaneswar, Odisha, India

**Keywords:** Chikungunya, anti-viral, infection, inflammation, replication

## Abstract

The increase in disease incidences and persistent Chikungunya virus (CHIKV)-induced arthritis have been a huge burden on public health globally. In the absence of specific antivirals or vaccines, it is essential to continue efforts to develop effective anti-CHIKV strategies. Our previous study showing the *in vitro* anti-CHIKV potential of a novel molecule 1-[(2-methylbenzimidazol-1-yl) methyl]-2-oxo-indolin-3-ylidene] amino] thiourea (MBZM-N-IBT) encouraged us to further validate its efficacy. Here, the effect of MBZM-N-IBT was evaluated *in vitro* in RAW 264.7 cells, *in vivo* in C57BL/6 mice, and *ex vivo* in human peripheral blood mononuclear cells (hPBMCs). The study demonstrated that CHIKV infection was efficiently abrogated in RAW 264.7 cells (IC_50_ = 22.34 μM) with significant inhibition in viral proteins. The inhibition was effective in the postentry step, and MBZM-N-IBT predominately interfered in the early stages of CHIKV life cycle. It was further supported when the protease activity of CHIKV-nsP2 was hindered by the compound. Moreover, it diminished the CHIKV-induced inflammatory responses *in vitro* through significant downregulation of all the major mitogen-activated protein kinases (MAPKs), NF-κB, cyclooxygenase (COX)-2, and cytokines. Furthermore, MBZM-N-IBT restricted CHIKV infection and inflammation *in vivo*, leading to reduced clinical scores and complete survival of C57BL/6 mice. Additionally, it has been noticed that the CHIKV infection was reduced remarkably in hPBMC-derived monocyte-macrophage populations *ex vivo* by the compound. In conclusion, it can be suggested that this novel compound MBZM-N-IBT has been demonstrated to be a potential anti-CHIKV molecule *in vitro*, *in vivo*, and *ex vivo* and fulfilled all the criteria to investigate further for successful treatment of CHIKV infection.

## INTRODUCTION

Chikungunya virus (CHIKV) was first identified in Tanzania (1952) (1). It caused sporadic outbreaks in tropical/subtropical regions of the world (Africa, surrounding locations in the Indian Ocean, and India) during first 50 years of its identification (2). According to a World Health Organization (WHO) fact sheet (https://www.who.int/news-room/fact-sheets/detail/chikungunya), since 2004, CHIKV has spread rapidly to more than 60 countries across the globe and become a global pathogen. As per the recent estimate of European Centre for Disease Prevention and Control, about 217,074 cases have been reported in 2021 across Asia, Africa, Americas, and the Caribbean (https://www.ecdc.europa.eu/en/publications-data/communicable-disease-threats-report-21-27-november-2021-week-47).

CHIKV is a vector-borne virus, mostly transmitted by female Aedes aegypti and Aedes albopictus mosquitoes (3). Sudden onset of fever and joint pain are the prominent symptoms of CHIKV infection (4). In addition, it is associated with symptoms common in viral infections, including muscle pain, joint swelling, headache, nausea, fatigue, and rash (5). While the febrile illness is often self-limiting, the joint pain is debilitating and may persist for months or even years. This leads to significant morbidity with adverse socioeconomic consequences. Further, it can affect the central nervous system (CNS) to aggravate the morbidity (6). Although the mortality rate is considered to be relatively low, pre-existing morbidity can increase mortality. Thus, more than 35,000 deaths have been related to CHIKV infection in the Americas and the Caribbean during 2014 to 2017 (7). Despite efforts, no licensed commercial vaccine or antiviral is available to treat this infection (8). Therefore, there should be continuous efforts to develop antiviral strategies against CHIKV infection.

CHIKV is a spherical shaped (70 nm), enveloped, RNA virus belonging to the Alphavirus genus of the Togaviridae family (9). The 11.8-kb-long single-stranded positive sense (+SS) (10) genome transcribes into two transcripts that further translates nonstructural (nsP1, 2, 3, and 4) and structural (C, E1, E2, E3, and 6k) poly proteins (11). Mature nonstructural and structural proteins are generated by the precise proteolytic cleavage of these polyproteins. The protease activity of the nsP2-carboxy terminus (CT) is critical for nonstructural protein processing (12–15). Post-transcriptional modification of nsP2 affects its protease activity (16). Apart from this, nsP2 is also responsible for the host-mediated transcriptional shutoff (17) and the downregulation of different viral restriction factors (18). Thus, the nsP2 protein is considered an important drug target for developing antiviral for CHIKV (19, 20). Earlier, 1-[(2-methylbenzimidazol-1-yl) methyl]-2-oxo-indolin-3-ylidene] amino] thiourea (MBZM-N-IBT) was found to reduce the nsP2 protein level by 97%, while the reduction in the RNA synthesis for nsP2 was only 65%. Based on this relatively remarkable inhibition of the nsP2 protein level and *in silico* analysis that showed possible interaction with the nsP2 protease domain (nsP2-CT), MBZM-N-IBT was proposed as a possible inhibitor of nsP2 (21). Thus, this study was undertaken to validate its ability to inhibit nsP2 protease activity. Further, MBZM-N-IBT showed good selectivity (CC_50_/IC_50_ > 21) in Vero cells for anti-CHIKV effect (21). The monocyte macrophage cell line, RAW264.7 is one of the major immune cells that is generally infected by the viruses and is shown to induce inflammation; thus, it is a more relevant *in vitro* model, and it is necessary to validate the antiviral activity in this cell line (22). It is also worthwhile to validate the efficacy *in vivo* and *ex vivo.* Hence, the efficacy of MBZM-N-IBT against CHIKV infection was investigated in a suitable preclinical mice model, as well as in human peripheral blood mononuclear cells (hPBMCs).

## RESULTS

### MBZM-N-IBT inhibits CHIKV infection efficiently in mouse monocyte/macrophage (RAW 264.7) cells.

**FIG 1:**
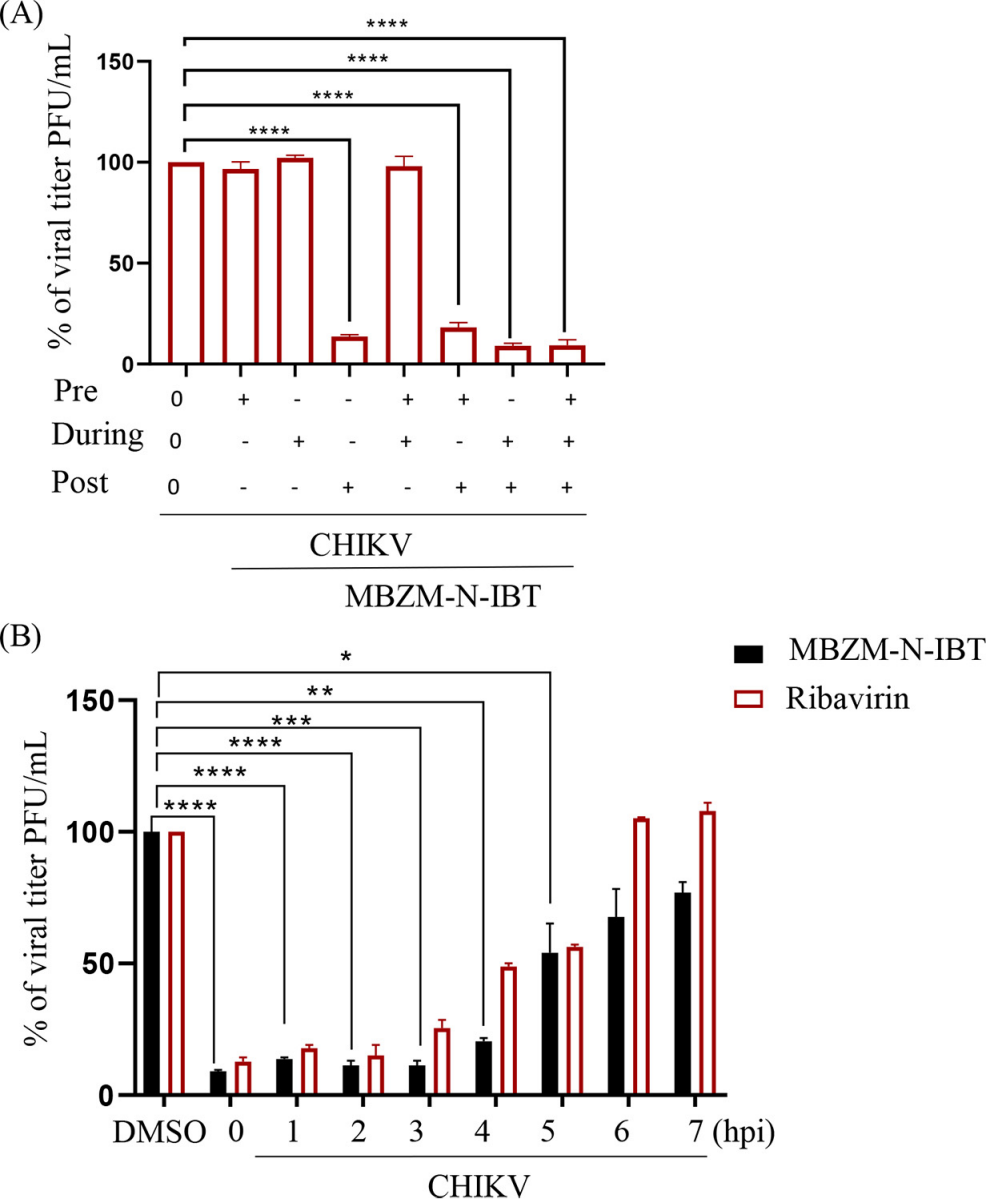
Post-treatment of 1-[(2-methylbenzimidazol-1-yl) methyl]-2-oxo-indolin-3-ylidene] amino] thiourea (MBZM-N-IBT) reduces Chikungunya virus (CHIKV) infection significantly. (A) The RAW 264.7 cells were treated with the compound (100 μM) separately (3 h before infection), during (1.5 h during infection), and after infection (8 h after infection). Compound was present before and during for “pre + during” conditions. Compound was present before, during, and after infection for “pre + during + post” infection. Supernatants collected at 8 h postinfection (hpi) were subjected to plaque assay. The bar diagram shows the percentage of virus particle. The data are presented as means ± standard error of the mean (SEM) (*n* = 3, *P ≤ *0.05 was considered statistically significant). (B) The RAW 264.7 cells were infected by CHIKV with a multiplicity of infection (MOI) of 5, and 100 μM compound was added to each sample, every 1-h interval up to 7 hpi. The open bars represent the viral titer obtained using ribavirin (10 μM) as a control. The bar diagram represents the percentage of virus titers of the supernatants collected at 8 hpi for all the samples. DMSO, dimethyl sulfoxide. *, *P* ≤ 0.05; **, *P* ≤ 0.01; ***, *P* ≤ 0.001; and ****, *P* ≤ 0.0001 were considered statistically significant.

To find out the cytotoxicity of MBZM-N-IBT, 3-(4,5-dimethyl-2-thiazolyl)-2,5-diphenyl-2H-tetrazolium bromide (MTT) assay was performed in RAW 264.7 cells. The viability of the RAW 264.7 cells, at 200 μM concentration of MBZM-N-IBT was found to be >80.3% (Fig. S1A). Thus, a concentration of 100 μM or less was used. The IC_50_ was found to be 22.34 μM (Fig. S1B) against CHIKV-PS in RAW 264.7 cells. This anti-CHIKV capability was also very prominent from a subsequent confocal microscopy that showed a dose-dependent reduction of viral antigen E2 (Fig. S1C and D). Hence, the data suggest that this molecule has significant capacity to inhibit CHIKV infection in monocyte/macrophage (RAW 264.7) cells *in vitro*. Virus-infected and compound-treated RAW 264.7 cell lysates were subjected to Western blot analysis to validate the effect of the compound on CHIKV structural (E2) and nonstructural (nsP2) proteins. The nsP2 and E2 protein levels were reduced by 88% and 94%, respectively, at a 100 μM concentration (Fig. S1E to G). However, significant reduction was also documented for other nsPs (Fig. S2A to D). Thus, it could be suggested that MBZM-N-IBT inhibits CHIKV infection. Furthermore, it has been observed that the compound has no antiviral effect during viral attachment and entry process; nonetheless, the best inhibitory effect was found during active propagation of the virus (Fig. 1A). Moreover, the “time of addition” experiment in RAW 264.7 cells has indicated that the compound is more effective in early phases of the CHIKV life cycle (Fig. 1B).

### MBZM-N-IBT interacts with CHIKV-nsP2-CT and reduces its protease activity.

**FIG 2:**
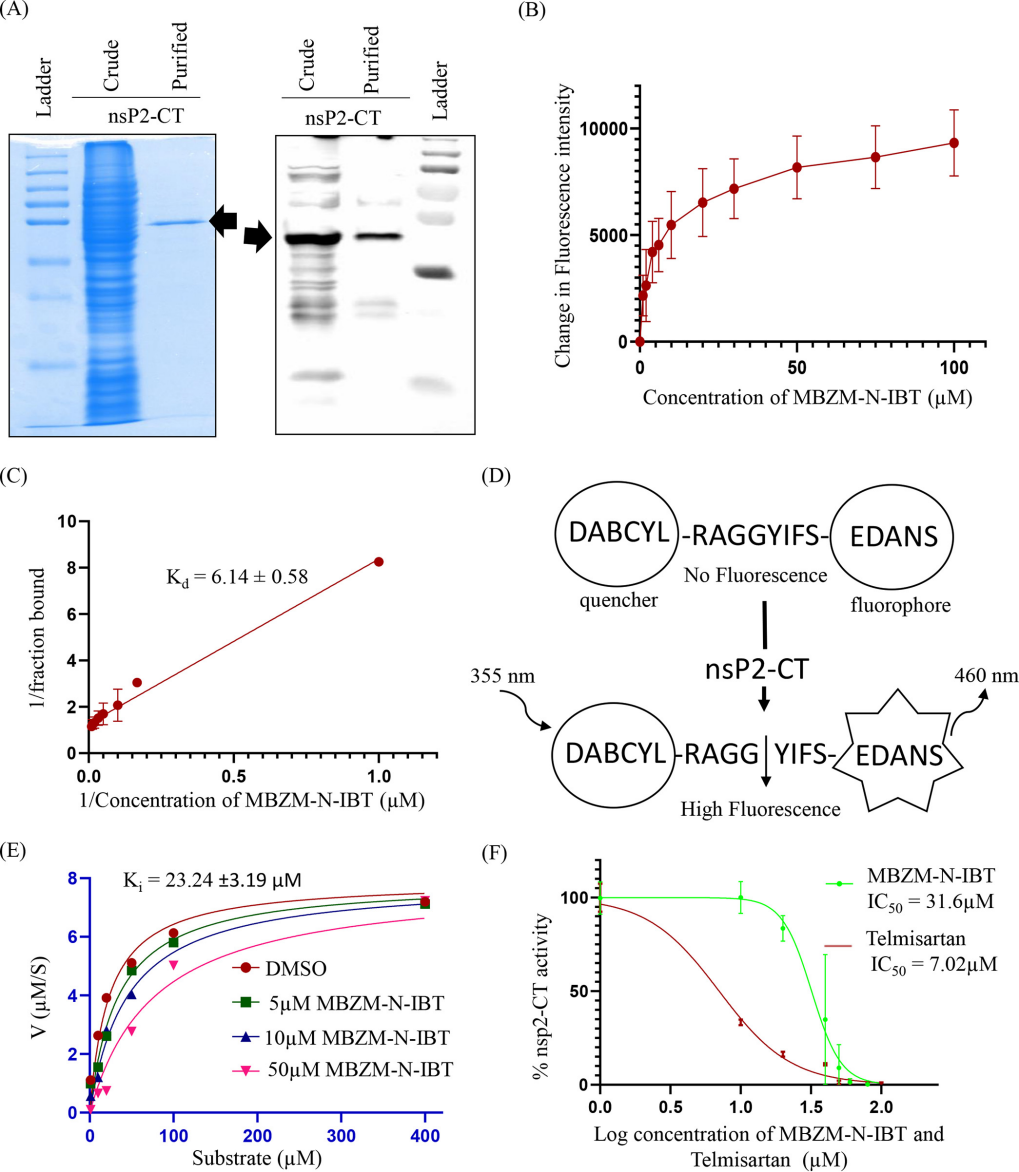
MBZM-N-IBT interacts with CHIKV-nsP2-CT and reduces its protease activity. (A) Crude and purified nsP2-CT (47 kDa) was resolved with SDS-PAGE under reducing conditions and subjected to Coomassie blue staining (left panel) and Western blot analysis (right panel) by nsP2-CT-specific antibody. (B) 1 μM nsP2-CT was incubated with or without increasing concentration (1, 2, 4, 6, 10, 20, 30, 50, 75, and 100 μM) of compound. The intrinsic tryptophan emission intensity was measured at 335 nm, and a gradual reduction in fluorescent intensity was observed. The graph shows binding of MBZM-N-IBT to nsP2-CT. (C) The dissociation constant (*K_d_*) for the interaction between the compound and nsP2-CT was derived by the double reciprocal plot. (D) Schematic diagram illustrating the principle of the fluorescence resonance energy transfer (FRET) assay. The cleavage site present between nonstructural proteins (nsP3-nsP4) of CHIKV (RAGGYIFS) is inserted between the fluorescence quenching pair (4-(dimethylaminoazo) benzene-4-carboxylic acid [DABCYL] and 5-((2-aminoethyl) amino) naphthalene-1-sulfonic acid [EDANS]. Cleavage of the peptide bond at RAGG*YIFS by the nsP2-CT proteins separates DABCYL and EDANS. Enhanced fluorescence can be monitored at 460 nm with excitation at 355 nm. (E) Kinetic parameters of nsP2-CT with or without increasing concentrations of compound were determined by fitting untransformed data into the Michaelis-Menten equation using GraphPad Prism software. (F) The percentage of enzymatic activity was calculated as a function of inhibitor concentration, and the IC_50_ value of the compound is represented in this graph obtained through GraphPad Prism software. Telmisartan was used as a positive control. All the experiments were performed in duplicate. Values from three independent experiments were used to obtain all the graphs.

To determine the interaction between MBZM-N-IBT with nsP2-CT, the purified nsp2-CT (Fig. 2A) protein (1 μm) was titrated against different concentrations of MBZM-N-IBT. The tryptophan fluorescence intensity of nsP2-CT was measured for an emission range of 310 to 370 nm, while the sample was excited at 290 nm. The change in the fluorescence intensity at 335 nm (Fig. 2B) was used to calculate the *K_d_* value using double reciprocal plot (Fig. 2C). The dissociation constant (*K_d_*) of MBZM-N-IBT to nsP2-CT was found to be 6.14 ± 0.58 μM, by preforming three independent experiments (Fig. 2C). A fluorescence resonance energy transfer (FRET)-based assay was performed to estimate the protease activity of CHIKV-nsP2-CT. A peptide substrate (DABCYL-DRAGG*YIFSE-EDANS) containing the cleavage site of the nsP3-nsP4 of CHIKV was used to monitor the *in vitro* protease activity (23). In this peptide, EDANS [5-((2-aminoethyl) amino) naphthalene-1-sulfonic acid] is a fluorophore, and DABCYL (4-(dimethylaminoazo) benzene-4-carboxylic acid) is a quencher. When the CHIKV-nsP2-CT hydrolyzes the substrate to yield two fragments, DABCYL is separated with EDANS, which relieves the fluorescence quenching effect, resulting in an increase in fluorescence signal (Fig. 2D). The initial velocity of the protein at different substrate concentrations was determined to calculate the kinetic parameters. The fluorescent extinction coefficient (FEC) of the FRET substrate was found to be 2145 RFU/μM. According to GraphPad Prism software, untransformed data were plotted and fitted to the Michaelis-Menten equation to determine various kinetic values for the given peptide substrate. The *K_m_* was calculated to be 14.79 ± 1.85 μM, and *V*_max_ was 7.10 ± 0.22 μM s^−1^, while *k*_cat_ and *k*_cat_/*K_m_* were calculated to be 4.73 ± 0.14 s^−1^ and 0.33 ± 0.32 μM^−1^ s^−1^, respectively, for the CHIKV-nsP2-CT protein (Fig. 2E). Further, different concentrations of the compound were tested to determine the inhibitory effect with increasing substrate concentrations. The decreased fluorescence intensity with the increasing concentrations of the compound validated the inhibition of CHIKV-nsP2-CT activity, and the data were fitted to Michaelis-Menten equation in GraphPad Prism software, and based on the best fit model, the inhibitor constant *K_i_* was calculated to be 23.24 ± 3.19 μM (Fig. 2E). Further, the IC_50_ values were 31.96 and 7.02 μM for MBZM-N-IBT and telmisartan, respectively, from the graphs plotted (log concentration versus normalized response-variable slope) in GraphPad Prism software where telmisartan was used as positive control (24) (Fig. 2F). All values are the means ± SD for three independent experiments, and the data indicate that MBZM-N-IBT interacts with CHIKV-nsP2-CT and reduces its protease activity.

### MBZM-N-IBT reduces the CHIKV-induced upregulation of MAPKs pathway, NF-κB, and COX-2 in RAW 264.7 cells.

**FIG 3:**
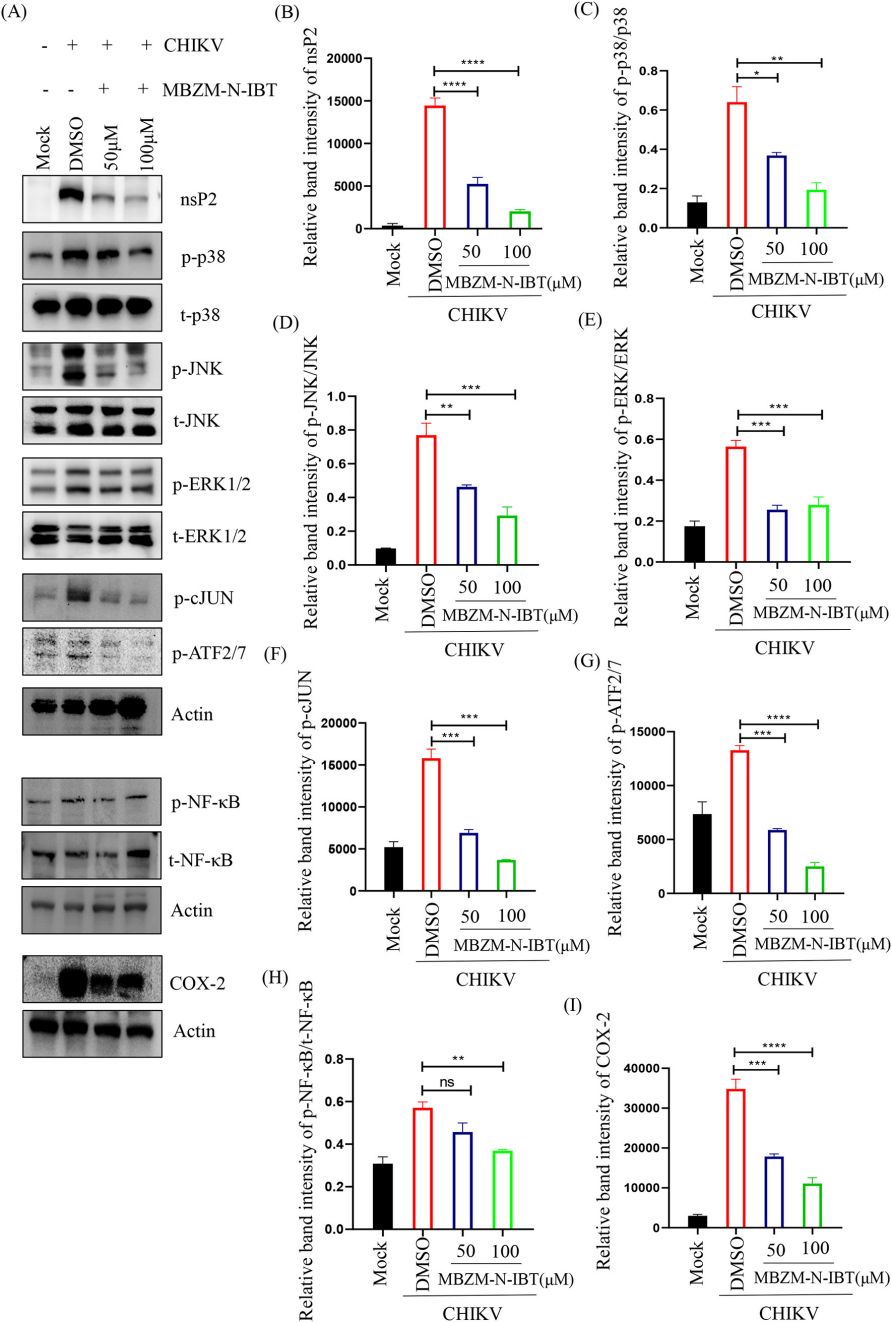
MBZM-N-IBT reduces the (CHIKV-induced) upregulated of mitogen-activated protein kinases (MAPKs), NF-κB, and COX-2 in RAW 264.7 cells. The RAW 264.7 cells were infected with CHIKV, treated with two different concentrations of MBZM-N-IBT (50 and 100 μM), and harvested at 8 hpi. (A) Western blot showing the nsP2 protein level along with phosphorylation status of p38, JNK, extracellular signal-regulated kinase (ERK), cJUN, ATF2/7, NF-κB, and total expression levels of COX-2 in RAW 264.7 cell lysates. Actin was used as a loading control. (B to H) Bar diagrams indicating relative band intensities of nsP2, phosphorylated (p)-p38, p-JNK, p-ERK, p-cJUN, p-ATF2/7, and p-NF-κB. (I) Bar diagram depicting relative band intensities of COX-2 protein. The data represent the means ± SEM (*n* = 3). *P ≤ *0.05 was considered statistically significant.

In order to examine CHIKV-induced activation of p38, extracellular signal-regulated kinase 1/2 (ERK1/2), Jun N-terminal kinase (JNK), cellular Jun proto-oncogene (cJUN), cyclic AMP-dependent transcription factor 2/7 (ATF2/7), nuclear factor κ-light-chain-enhancer of activated B cells (NF-κB), and COX-2 by MBZM-N-IBT, a Western blot experiment was performed using mock, virus-infected, and compound-treated RAW 264.7 cell lysates. Significant activation in the phosphorylation of each of the leading mitogen-activated protein kinases (MAPKs): p38 (6-fold), JNK (8-fold), and ERK1/2 (6-fold) were observed after CHIKV infection (Fig. 3A and B). However, phosphorylation of p38, JNK, and ERK1/2 were reduced by 3-, 2.5-, and 2-fold, respectively, following treatment (Fig. 3A, C, D and E). Since phosphorylated cJUN and ATF2/7 are the major downstream transcription factors for the expression of proinflammatory cytokines through the MAPK pathway (25, 26), they were estimated, and it was demonstrated that inductions of phosphorylated (p)-cJUN and p-ATF2/7 were 4- and 2-fold higher after infection, respectively, whereas MBZM-N-IBT treatment reduced the level of p-cJUN by 5-fold and p-ATF2/7 by 4.5-fold compared to infection (Fig. 3A, F and G). Similarly, infection increased the level of p-NF-κB (p65) by 2-fold, which was downregulated (3-fold) by the compound (Fig. 3A and H). Because COX-2 is a key enzyme that is overexpressed during inflammation (27), its level was estimated following infection and treatment; although its level was higher by 10-fold, the same was downregulated by MBZM-N-IBT (3-fold) (Fig. 3A and I). Altogether, MBZM-N-IBT significantly downregulated the CHIKV-induced activation of all major MAPKs, NF-κB, and COX-2.

### MBZM-N-IBT reduces the CHIKV-induced proinflammatory cytokine production in RAW 264.7 cells.

To investigate the role of MBZM-N-IBT on the CHIKV-induced inflammatory cytokine and chemokine production, mock, virus-infected, and compound-treated RAW 264.7 cell supernatants were subjected to Milliplex assay. It has been found that CHIKV infection in RAW 264.7 cells caused elevated production of different proinflammatory cytokines and chemokines. However, a significant reduction in the production of all major proinflammatory cytokines (tumor necrosis factor [TNF]-α [30%], interleukin [IL]-1β [57%], IL-6 [52%], IL-12 [56%], granulocyte-macrophage colony-stimulating factor [GM-CSF] [52%], and granulocyte colony-stimulating factor [G-CSF] [43%]) were observed after compound treatment (Fig. S3A to F). In addition to that, the levels of different chemokines (IP10 [CXCL-10 (IFN-γ-inducible protein 10)] [20%], MCP-1 [monocyte chemoattractant protein-1] [33%], MIP-1 [macrophage inflammatory protein-1] [15%], RANTES [CCL-5 (regulated on activation, normal T cell expressed and secreted)] [25%], and KC [CXCL-1 (keratinocytes-derived chemokine)] [40%]) were also reduced significantly (Fig. S3G to K). After that, the sole effect of the compound on the well studied key inflammatory and virus-restriction factors were assessed. For that, the compound was added to uninfected RAW 264.7 cells along with infected and treated samples. Then their RNA levels were estimated by reverse transcription-quantitative PCR (RT-PCR) (Table S1). Interestingly, no or insignificant fold changes of the different immunomodulatory host factors (interferon [IFN]-β, TNF-α, MCP-1, and 2'-5'-oligoadenylate synthetase 1 [OAS1]) were observed when the cells were treated with the compound only (Fig. S4A to E). However, the compound significantly downregulated these host factors when they were upregulated following CHIKV infection (Fig. S4A to E). Together, the data indicate that MBZM-N-IBT reduces the CHIKV-induced proinflammatory cytokine production without affecting their levels in normal host cells.

### MBZM-N-IBT protects mice from CHIKV infection by diminishing the overall viral burden, viral RNA, and protein levels.

**FIG 4:**
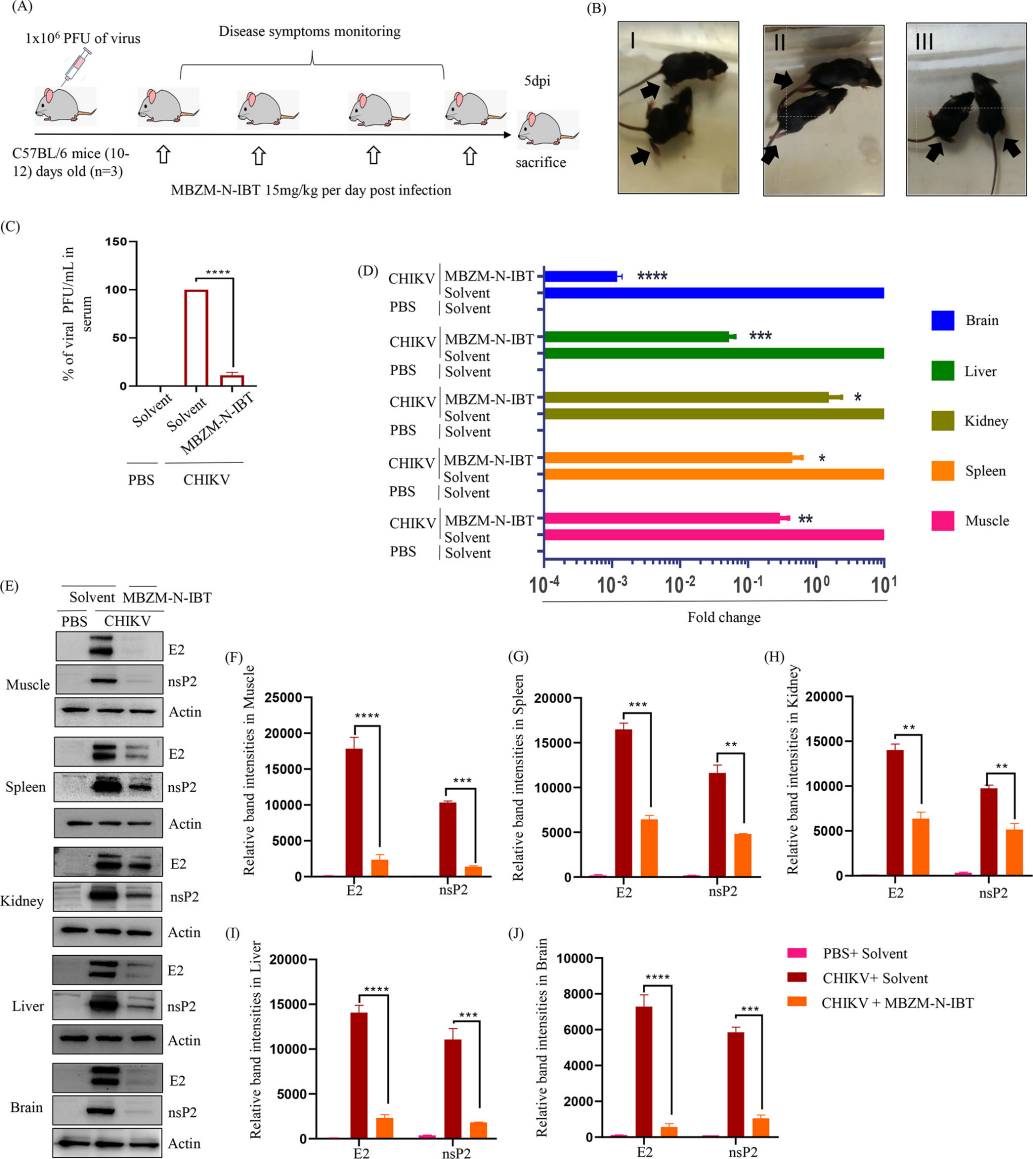
MBZM-N-IBT protects mice from CHIKV infection by diminishing the overall viral burden, viral RNA, and protein levels. (A) C57BL/6 mice were infected subcutaneously with 10^6^ PFU of CHIKV and treated with 15 mg/kg MBZM-N-IBT at 24-h intervals up to 4 days postinfection (dpi). The mice were sacrificed at 5 dpi. Sera and different tissues were also collected for further downstream experiments. (B) Image of mock, CHIKV-infected, and compound-treated mice. (C) Equal amount of muscle tissue was taken and homogenized followed by filtration (0.22 μm). Homogeneous sample was subjected to plaque assay. The bar diagram shows CHIKV percentage of PFU/mL in virus-infected and compound-treated mice serum. (D) Whole RNA was isolated from the CHIKV-infected and compound-treated samples, and the CHIKV E1 gene was amplified by reverse transcription-quantitative PCR (qRT-PCR). The bar diagram shows the fold change of viral RNA in infected and compound-treated samples. (E) Western blot showing the viral E2 and nsP2 protein in different tissue samples. Actin was used as a loading control. (F to J) Bar diagrams showing the relative band intensities of E2 in infected and compound-treated samples of different tissues. All bar diagrams were obtained through GraphPad Prism software (*n* = 3). The data are presented as means ± SEM (*P ≤ *0.05 was considered statistically significant). PBS, phosphate-buffered saline; plaque-forming units per milliliter (PFU/mL).

The anti-CHIKV effect of MBZM-N-IBT was assessed in 10- to 12-day-old C57BL/6 mice infected with CHIKV following treatment with increasing concentrations of compound (5, 10, 15, 25, and 50 mg/kg) at 24-h intervals up to 5 days postinfection (dpi) (Fig. 4A). After that, the viral titer was monitored, and a 15 mg/kg dose was found to be optimal for further studies (Fig. S5). The infected group of mice showed symptoms of impaired mobility due to arthritis in limbs, whereas compound-treated animals had no such abnormalities (Fig. 4B). The infected and compound-treated mice were sacrificed on 5 dpi. CHIKV particles were found to be reduced by 93% following treatment in mice serum (Fig. 4C). After that, viral RNA and protein levels were also estimated in different tissues. In muscles, remarkable reduction in viral RNA (E1: 10-fold) (Fig. 4D) and proteins (E2: 12-fold; nsP2: 10-fold) (Fig. 4E and F) were observed. Similarly, reduction of viral RNA was also detected in other organs like the liver (100-fold), kidney (2-fold), and spleen (100-fold) (Fig. 4D). Moreover, the CHIKV-E2 and CHIKV-nsP2 protein levels were also reduced in spleen (E2: 3-fold; nsP2: 2.5-fold) (Fig. 4E and G), kidney (E2: 3-fold; nsP2: 2-fold) (Fig. 4E and H), liver (E2: 6-fold; nsP2, 10 fold) (Fig. 4E and I), and brain (E2: 15-fold; nsP2: 8-fold) (Fig. 4E and J). Collectively, these results suggest that MBZM-N-IBT has the potential to protect mice from CHIKV infection.

### MBZM-N-IBT treatment dampens the induction of proinflammatory cytokines that are upregulated by CHIKV infection in mice.

To investigate whether MBZM-N-IBT treatment modulates the cytokine response *in vivo*, virus-infected and compound-treated mice serum was subjected to Milliplex assay. For this, the mice were infected and treated according to the previously mentioned protocol. As cytokine response is very dynamic in *in vivo* system, mice from each group were sacrificed, and serum was collected every day postinfection (1 to 5 dpi). CHIKV infection in C57BL/6 mice led to increased production of proinflammatory cytokines and chemokines (TNF-α, IL-2, KC, IL-6, GM-CSF, IP-10, MIP-1β, IL-1β, IL-12, IFN-γ, macrophage colony-stimulating factor [M-CSF], MCP-1, CXCL-9 (monokine induced by γ interferon) [MIG], and RANTES) that have been associated with disease severity, morbidity, and mortality in CHIKV-infected patients (28). Among these cytokines and chemokines, both TNF-α and IL-2 were significantly less in treated groups throughout the study (Fig. S6A and B). For KC, a considerable difference was noticed until the 4 dpi (Fig. S6C). Up to 3 dpi, induction of IL-6, GM-CSF, IP-10, and MIP-1β was suppressed (Fig. S6D to G). Interestingly, the production of IL-1β, IL-12, IFN-γ, M-CSF, MCP-1, MIG, and RANTES were downregulated only for the initial days of the study (1 or 2 dpi) (Fig. S6H to N). In addition to that, viral titer was also estimated at every dpi, and it was found that there was remarkable reduction in the treated group throughout the study (Fig. S6O). Apart from that, the status of other different inflammatory markers were also investigated. For this, the serum collected on 5 dpi was subjected to proteome profiler assay. Upregulation of all markers were found in infected mice. However, in the treated group, they all (complement factor D, C-reactive protein, amphiregulin, pentraxin 3, Gas6, ICAM-1 [CD54], VCAM-1 [CD106], E-Selectin) were significantly downregulated (Fig. S7A and B). Thus, MBZM-N-IBT treatment has beneficial roles in the resolution of CHIKV-induced inflammation *in vivo*.

### MBZM-N-IBT decreases the swelling of muscle and spleen and improves their histopathology in CHIKV-infected mice.

**FIG 5:**
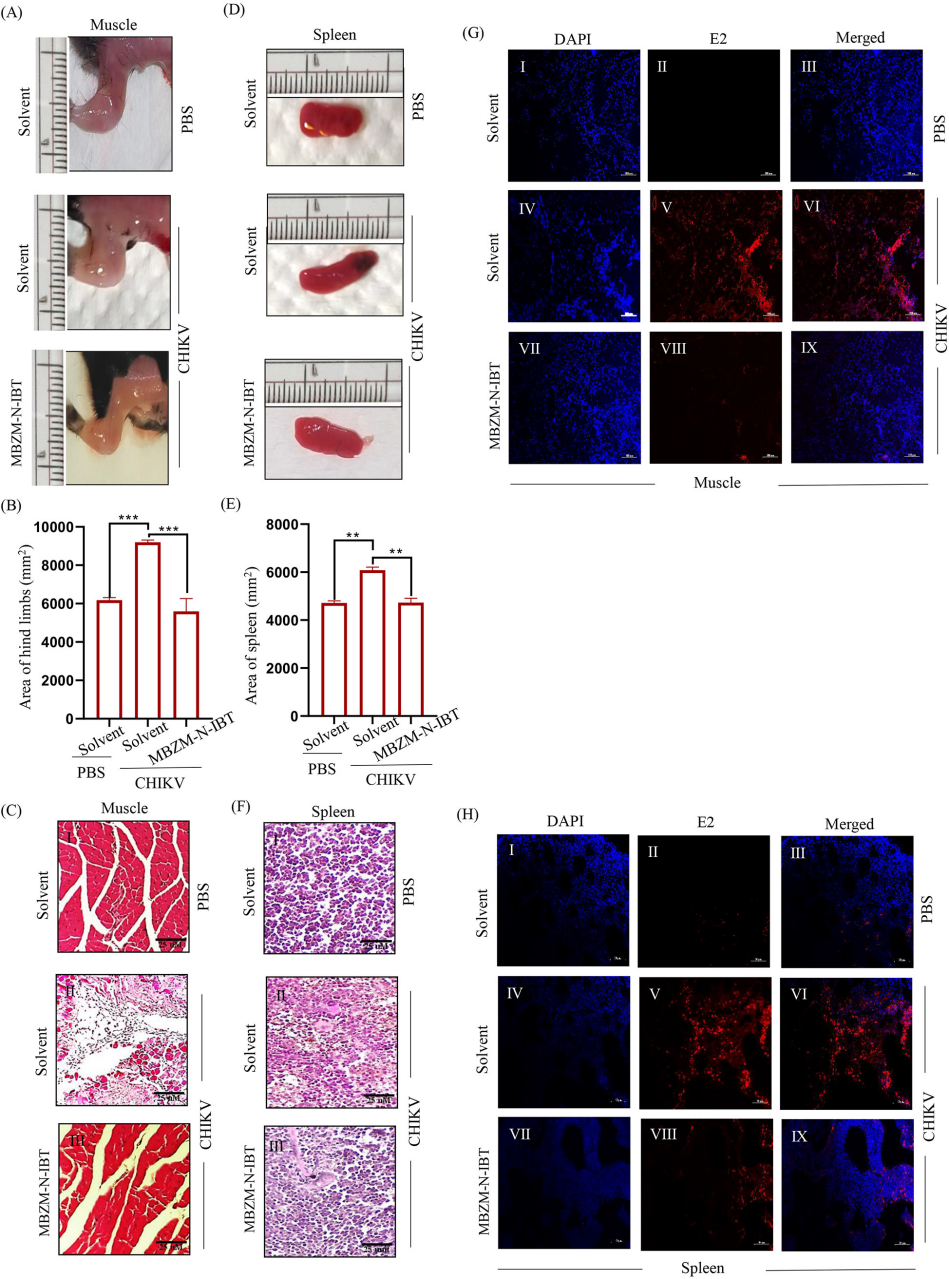
MBZM-N-IBT decreases the swelling of muscle and spleen and improves their histopathology in CHIKV-infected mice. C57BL/6 mice were infected subcutaneously with 10^6^ PFU of CHIKV and treated with 15 mg/kg MBZM-N-IBT at 24-h intervals up to 4 dpi. The mice were sacrificed at 5 dpi. Muscle and spleen tissues were collected for morphological and histopathological analysis. (A) Image panels indicating evident hind limb muscle swelling of mock, infected, and treated groups of mice. (B) Bar diagram showing the quantitation of limb swelling. (C) Image panels showing the hematoxylin and eosin (H&E)-stained muscle section. (D) Image panels depicting gross swelling of spleen (splenomegaly) of mock, infected, and treated groups of mice. (E) Bar diagram showing the quantitation of splenomegaly. (F) Image panels showing the H&E-stained spleen section. (G) Image panels showing the CHIKV-E2 stained muscle sections. (H) Image panels showing the CHIKV-E2 stained spleen tissue sections. All bar diagrams were obtained through GraphPad Prism software (*n* = 3). The data are presented as the means ± SEM (*P ≤ *0.05 was considered statistically significant). DAPI, 4′,6-diamidino-2-phenylindole.

*In vivo* infection of CHIKV causes swelling of limb muscle and spleen (29). In order to quantitate the swelling, the total surface area of the infected hind limb muscle (*n* = 3) and spleen (*n* = 3) were measured using ImageJ software. The limb muscle (50%) and spleen (33%) were found to be swollen after CHIKV infection. Treatment with compound lowered this to a normal level (Fig. 5A, B, D and E). Furthermore, histological analysis was conducted by hematoxylin and eosin (H&E) staining and immunohistochemistry (IHC) to examine the histopathology of infected and treated muscle and spleen. H&E staining revealed that there was muscle necrosis and huge infiltration of mononuclear lymphocytes in the infected muscle section and its eventual reduction in the treated section (Fig. 5C). Interestingly, the presence of centrally located nuclei indicated muscle regeneration after treatment (Fig. 5C). Moreover, hyperplasia and accumulation of infiltrated fluid were observed in the H&E-stained section of the infected spleen that was reduced significantly upon treatment (Fig. 5F). Further, IHC was performed to detect the expression of viral antigen (CHIKV-E2) in infected tissues. While an intense E2 signal was detected in CHIKV-infected mice muscle (Fig. 5G) and spleen (Fig. 5H), treatment with MBZM-N-IBT prevented its overexpression. Altogether, these data suggest the potential of MBZM-N-IBT to manage CHIKV infection and related symptoms *in vivo*.

### MBZM-N-IBT treatment ameliorates CHIKV-induced disease score and provides better survival.

**FIG 6:**
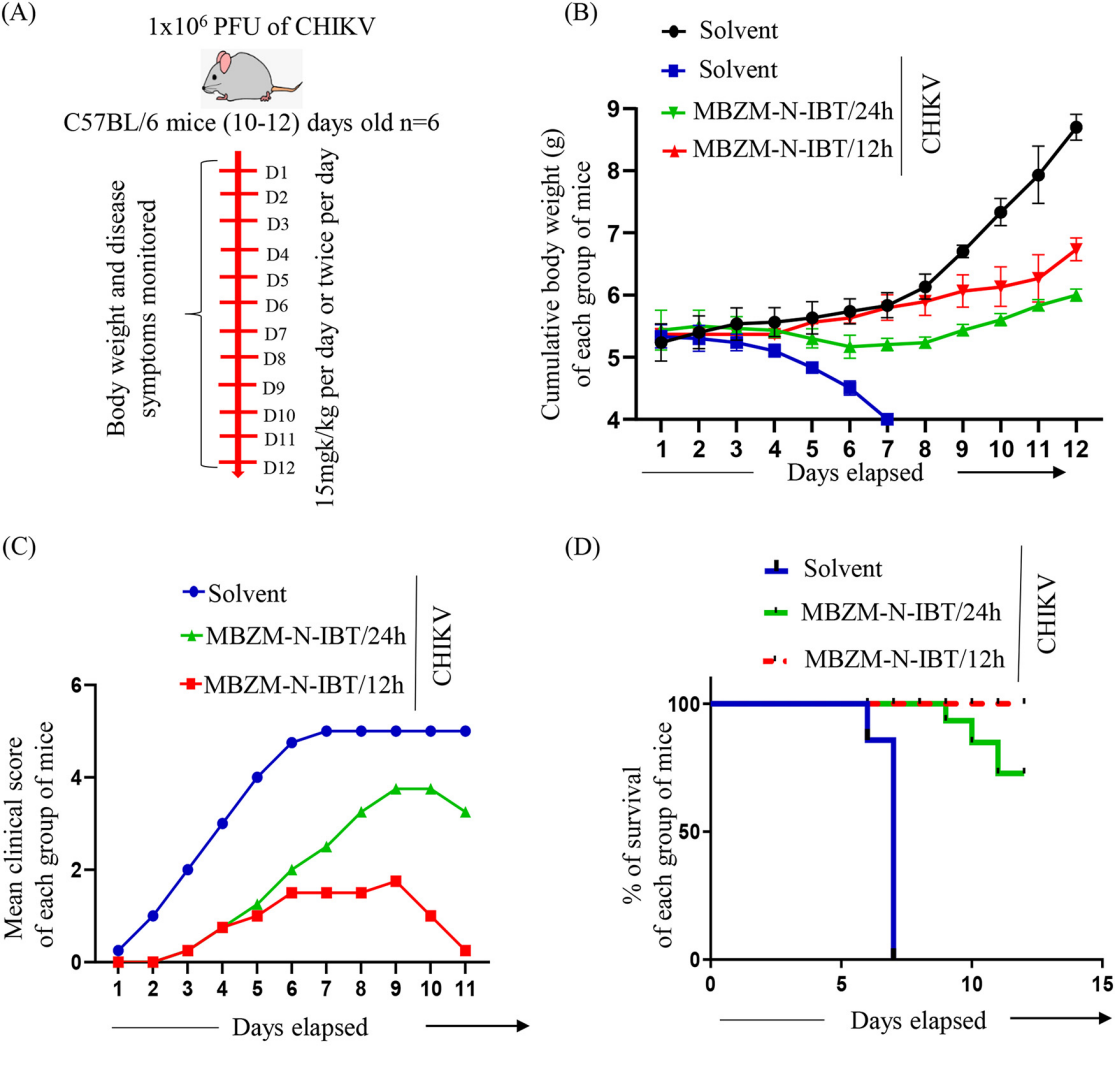
MBZM-N-IBT treatment ameliorates CHIKV-induced disease score and provides better survival. (A) C57BL/6 mice were infected subcutaneously with 10^6^ PFU of CHIKV and treated with 15 mg/kg MBZM-N-IBT at 24- or 12-h intervals up to 12 dpi. Six mice were taken in each group. (B) Graph showing cumulative body weight of each group of mice throughout the study. (C) Graph describing mean body weight of animals in each group at each dpi. (D) Line diagram showing the survivability of infected mice along with the treated groups. The percentage survivability data were obtained through GraphPad Prism software.

To determine the effect of MBZM-N-IBT on the overall disease outcome and survivability, compound-treated (15 mg/kg per 24 h and 15 mg/kg per 12 h) or untreated mice infected with 10^6^ PFU of CHIKV were monitored for the development of disease signs and mortality up to 12 dpi (Fig. 6A). While the untreated group exhibited severe weight loss from 1 dpi, the cumulative bodyweight of the treated group of mice remained stable up to 8 dpi and gradually increased after that (Fig. 6B). Infection resulted in severe disease score in untreated groups, and all the CHIKV-infected mice died on 7 dpi. However, the, treatment with MBZM-N-IBT minimized the disease score significantly (Fig. 6C). A dose of 15 mg/kg (once per day) provided 86% survival, whereas an increase in the dosing frequency (twice per day) provided 100% protection (Fig. 6D). These results support the *in vivo* efficacy of MBZM-N-IBT to ameliorate the disease conditions and to increase the survival of the mice.

### MBZM-N-IBT reduces CHIKV infection in hPBMC-derived monocyte-macrophage populations *ex vivo*.

**FIG 7:**
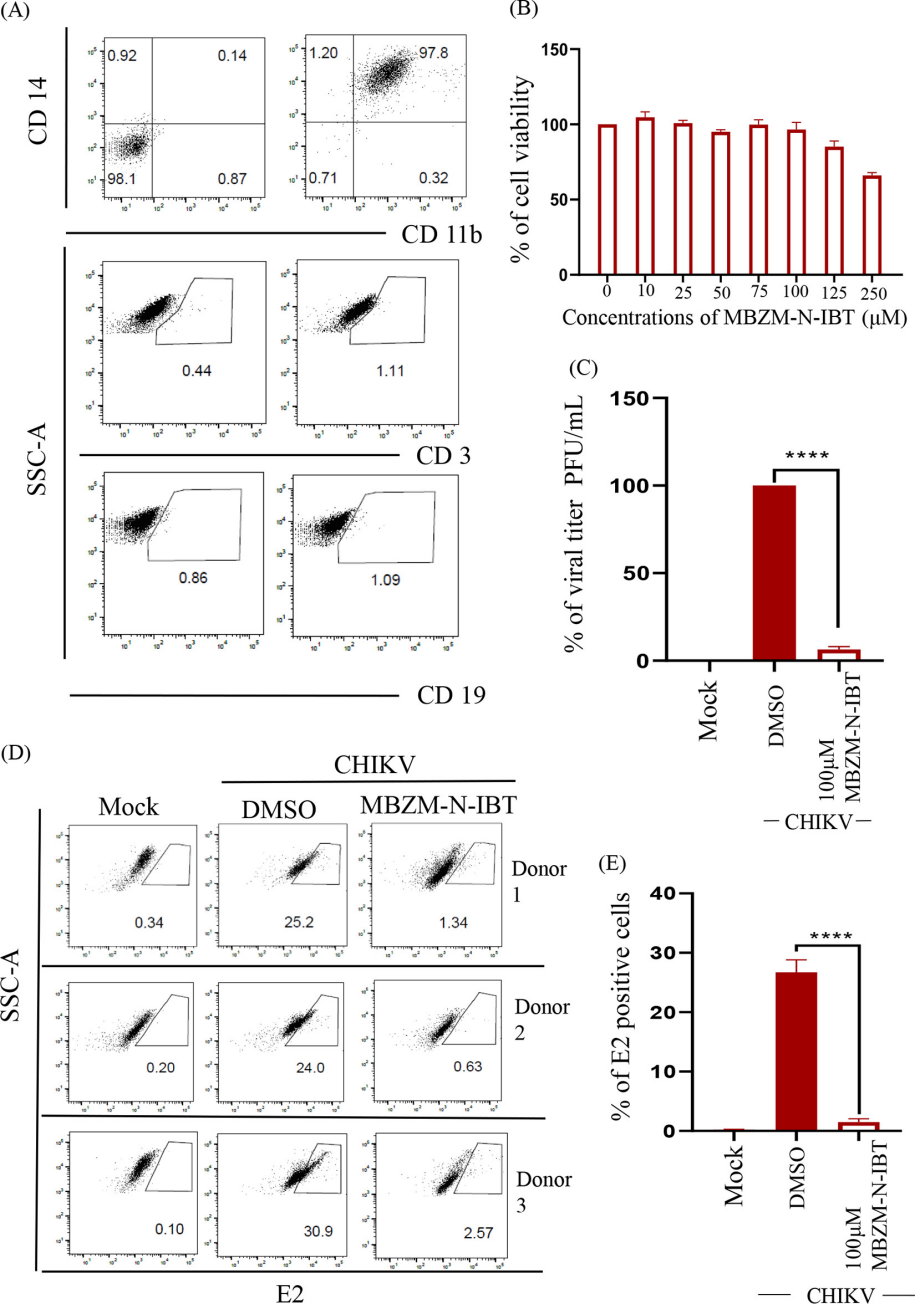
MBZM-N-IBT reduces CHIKV infection in human peripheral blood mononuclear cell (hPBMC)-derived monocyte-macrophage populations *ex vivo*. (A) Dot plot showing the percentages of CD14^+^CD11b^+^ monocyte-macrophage lineage of adherent hPBMCs, B cells (CD19), and T cells (CD3) by flow cytometry. (B) Bar diagram referring the cytotoxicity of MBZM-N-IBT in hPBMC-derived adherent population by 3-(4,5-dimethyl-2-thiazolyl)-2,5-diphenyl-2H-tetrazolium bromide (MTT) assay. (C) Bar diagram depicting percentage of the viral particle formation by plaque assay. (D) Dot plot showing the percentage of viral E2 positive monocyte-macrophage population in mock, CHIKV-infected, and compound-treated samples from three healthy donors’ hPBMCs by flow cytometry. (E) Bar diagram showing the percentage of positive cells for the CHIKV-E2 protein. The data shown are represented as means ± SEM of three independent experiments. *, *P < *0.05. Side scatter (SSC), 0.15 M NaCl plus 0.015 M sodium citrate.

To evaluate the anti-CHIKV effect of MBZM-N-IBT in the higher order mammalian system, adherent monocyte-macrophage cells (CD14^+^/CD11b^+^ cells) from hPBMCs was used. For that, cells were stained with B cell (CD19), T cell (CD3), and monocyte-macrophage cell (CD11b and CD14) specific markers and analyzed by flow cytometry to characterize the hPBMCs (Fig. 7A). A 125 μM concentration of the compound was found nontoxic to the CD14^+^/CD11b^+^ cells (Fig. 7B), and the cells collected from three healthy individuals were subjected to CHIKV infection (multiplicity of infection [MOI] of 5) followed by treatment (100 μM). It was observed that the compound was able to abrogate viral infection by 98% in hPBMCs (Fig. 7C). The infected hPBMCs showed 26.7 ± 3.68% positivity for E2, whereas treatment led to the decrease of 1.49 ± 0.94% (Fig. 7D and E). Taken together, the results suggest that MBZM-N-IBT might reduce CHIKV infection remarkably in hPBMC-derived monocyte-macrophage populations *ex vivo*.

## DISCUSSION

The increase in disease incidences and persistent CHIKV-induced arthritis-like symptoms have been a huge burden on public health across the globe. In the absence of any specific antiviral for CHIKV, it is essential to continue efforts to develop an effective antiviral strategy against this virus (30, 31). The earlier findings that showed the potential of MBZM-N-IBT to inhibit CHIKV *in vitro* encouraged us to further validate its efficacy (21).

In the current study, the CHIKV infection was efficiently abrogated in the mice monocyte/macrophage cells (RAW 264.7) with significant inhibition in the viral protein levels. The inhibition was effective in the postentry step of the virus, and MBZM-N-IBT predominately interfered in the early stages of CHIKV life cycle. It was further confirmed when the protease activity of CHIKV-nsP2 protein was hindered by the compound. Moreover, it diminished the CHIKV-induced inflammatory responses *in vitro* through significant downregulation of all the major MAPKs, NF-κB, COX-2, and cytokines that were elevated following CHIKV infection. Furthermore, MBZM-N-IBT restricted CHIKV infection and inflammation *in vivo*, leading to reduced clinical score and complete survival of C57BL/6 mice. Additionally, it was noticed that the CHIKV infection was reduced remarkably in hPBMC-derived monocyte-macrophage populations *ex vivo* by this compound.

Monocytes/macrophages are the first subpopulation of immune cells, which respond to viral infection with an elevated expression of proinflammatory cytokines (22). Although this response aims to mitigate the infection, it contributes to the inflammatory disorders associated with CHIKV infection (32). In the current investigation, the molecule showed an IC_50_ of 22.34 μM in CHIKV-infected RAW 264.7 cells (Fig. S1B). In last few years, harringtonine, silymarin, berberine, abamectin, ivermectin, and several other compounds were identified to inhibit CHIKV replication by reducing the level of CHIKV-nonstructural proteins (33, 34). In contrast, arbidol, suramin, amantadine, and picolinic acid target viral structural proteins to inhibit entry or attachment (33, 34). Although the effect of the compound was less during viral attachment and entry, maximum inhibition was observed during active propagation of the virus (Fig. 1A). However, the molecule was able to restrict the infection even if it was added toward the end of infection, indicating its role at the late phase of viral infection, which also needs further investigation.

The multifunctional nsP2 of CHIKV is an attractive target for antiviral development. In agreement with the earlier report (24), telmisartan showed an IC_50_ of 7.02 μM for nsP2 protease activity (Fig. 2F). Compared to this, the potency of MBZM-N-IBT against this target is relatively low, as 50% inhibition in nsP2 protease activity was found at 31.96 μM concentration. Nevertheless, the study reveals moderate binding affinity (*K_d_* value 6.14 ± 0.58 μM) (Fig. 2C) for the nsP2-CT while demonstrating significant inhibition of protease activity. Thus, the nsP2 protein can be considered one of the targets of MBZM-N-IBT. Most of the reports associated with the development of several inhibitors against CHIKV-nsP2 were based on *in silico* prediction and with no biological validation (20, 35–38). Among them, ribostamycin sulfate, a FDA-approved compound, and E-64, a cysteine protease inhibitor, form utmost stable complexes at the active site of nsP2 protease (39), whereas *in silico* predictions along with the cell-free protease inhibition assays were used to identify CHIKV-nsP2 inhibitors by Das et al. (19). This was further optimized to achieve increased efficacy (40). Apart from that, 4-hydroxy-1-methyl-3-(3-morpholinopropanoyl) quinoline-2(1H)-one (QVIR), a quinoline derivatives showed *in vitro* anti-CHIKV activity with potential binding affinities toward CHIKV-nsP2 and E2 glycoprotein (41). Moreover, 1,3-thiazolbenzamide derivatives was found to inhibit the CHIKV-nsP2 protease activity directly along with the reduction of other nonstructural CHIKV protein levels *in vitro* (42). This molecule showed a mechanism of abrogation similar to that in the current findings.

Inflammatory response is the major reason of arthritis/morbidity associated with CHIKV infection. Expectedly, infection led to the induction of phosphorylation of proinflammatory upstream kinases and COX-2 (43), a key enzyme for conversion of arachidonic acid to prostaglandins. The MBZM-N-IBT molecule was shown to reduce all these proinflammatory mediators and transcription factors, establishing its anti-inflammatory role. This was also supported by the reduction in the levels of cytokines and chemokines. Interestingly, in normal host cells, MBZM-N-IBT did not affect the immunomodulatory host factors (IFN-β, TNF-α, MCP-1, and OAS1). However, several reports suggested that the pretreatment with type I interferon (IFN) signaling inducers (viperin, 50pppRNA, and its analogue M8) can control and prevent CHIKV infections (30). Agonists of the STING pathway were also known to limit alphavirus infection (44). Contrastingly, Src family kinase, serin/threonine-protein kinase C, and Pi3-Akt inhibitors were found to be inhibit CHIKV infection by modulating host signaling directly (45–47). The host generally controls infection by upregulating the above-mentioned pathways, and this can be a good prophylactic strategy. However, after infection, this mechanism become less effective as severe inflammation complicates the situation. From this point of view, MBZM-N-IBT was found to be excellent to manage the induced immune response following infection.

Since MBZM-N-IBT showed the ability to modulate diverse proinflammatory mediators, further validation was carried out *in vivo*. Most of the murine models of CHIKV display inflammation and macrophage infiltration only in the adjacent tissues at the site of inoculation, and no signs of systemic polyarthritis were observed (48), whereas in the current study the model showed CHIKV-induced arthritis-like symptoms, which are comparable to CHIKV disease pathogenesis in human. The molecule was able to demonstrate good effect to modulate this symptoms. Since CHIKV affects the brain, these findings are interesting to manage infection in brain also (49). These symptoms can indicate its possible side effects, like sedation; however, no such effect was observed in this study. Another CHIKV replication inhibitor, berberine, which is plant alkaloid, was shown to reduce CHIKV-induced inflammatory disease *in vivo* (50). Apart from that, there are few synthetic compounds targeting CHIKV-nsP4 (favipiravir, sofosbuvir, SNX-2112, and HS-10) and documented to have *in vivo* anti-CHIKV efficacy (34). This is the first report of a CHIKV-nsP2 inhibitor that is found to be effective in mice (C57B/L6) as well as in human PBMCs.

The mechanistic insight has been explored in this study; however, in-depth investigation is required to identify the role of MBZM-N-IBT in host pathway modulation in the future. Apart from that, a mutational study can be performed to map the interaction sits of nsP2-CT and the compound. In addition, the efficacy of this compound on nonhuman primate (NHP) models can also be assessed before clinical trials, if possible. In conclusion, this novel compound MBZM-N-IBT has been demonstrated to be a potential anti-CHIKV molecule *in vitro*, *in vivo*, and *ex vivo* and fulfilled all the criteria to investigate further for successful treatment of CHIKV infection.

## MATERIALS AND METHODS

### Virus and cells.

The CHIKV prototype strain (PS, accession no. AF369024.2) was a generous gift from M.M. Parida (Defense Research Development Establishment [DRDE], Gwalior, India). The Vero (African green monkey kidney epithelial cells) and RAW 264.7 (mouse monocyte/macrophage cells) cells were procured from NCCS (National Center for Cell Science), India. Dulbecco’s modified Eagle’s medium (DMEM) (PAN Biotech, Aidenbach, Germany) supplemented with 10% fetal bovine serum (FBS) (PAN Biotech, Aidenbach, Germany), along with gentamycin and penicillin-streptomycin (PAN Biotech, Aidenbach, Germany) was used to maintain Vero cells. RAW 264.7 cells were maintained in RPMI 1640 (Gibco RPMI 1640 GlutaMAX; Invitrogen, CA) supplemented with 10% FBS (Gibco FBS, Invitrogen, CA), penicillin-streptomycin, and gentamycin. All of the cells were maintained at 37°C with 5% CO_2_ in a humidified incubator (Heracell, Thermo Fisher, MA) (51, 52).

### Antibodies and drugs.

The anti-CHIKV-nsP2 monoclonal, anti-CHIKV-nsP2-CT, anti-CHIKV-nsP1, anti-CHIKV-nsP3, and anti-CHIKV-nsP4 polyclonal antibodies were developed by our group (53, 54). The anti-CHIKV-E2 monoclonal antibody was a kind gift from M.M. Parida (DRDE, Gwalior, India). The antibodies for p38, p-p38, JNK, p-JNK, ERK1/2, p-ERK1/2, p-cJUN, p-ATF2/7, p-NF-κB, NF-κB, and COX-2 were purchased from CST (MA). The actin antibodies were purchased from Sigma-Aldrich (MA). Telmisartan was a kind gift from Glenmark Life Sciences Ltd., (Ankleshwar, Gujrat, India). MBZM-N-IBT was synthesized by our group (21, 55).

### Cytotoxicity assay.

To determine the cytotoxicity of MBZM-N-IBT, an MTT assay was performed using the EZcount MTT cell assay kit (Himedia, Mumbai, India) in RAW 264.7 cells according to the manufacturer’s protocol. Approximately 10,000 numbers of cells were seeded in 96-well plates 24 h before the experiment. At 80% confluence, the cells were treated with increasing concentrations of compound. For this assay, at 12 h post-treatment, 10 μL MTT reagent (5 mg/mL) was added to the cells and incubated for 1 h at 37°C. Next, the medium was removed, and 100 μL of solubilization buffer was added followed by incubation at 37°C for 15 min to dissolve the formazan crystals. Finally, at 570 nm, the absorbance was measured using a multimode plate reader (Perkin-Elmer, MA) to determine the cellular cytotoxicity (56).

### Virus infection.

The RAW 264.7 cells were infected with PS at MOI 5 followed by a 90-min incubation with shaking every 10 to 15 min. After infection, the inoculum was replaced with fresh medium with different concentrations of drug for 8 h. Next, infected and MBZM-N-IBT-treated cells, as well as supernatants, were harvested at different hours postinfection (hpi) for estimating the levels of viral RNA, proteins, and viral titers according to the methods described earlier (57).

### Plaque assay.

Vero cells were seeded in 12-well plates and were infected with different dilutions of cell supernatants or tissue homogenate (mock, infected, and treated). After 90 min, the cells were washed with 1× phosphate-buffered saline (PBS) and overlaid with DMEM containing methylcellulose (Sigma, MA) followed by incubation for 4 to 5 days. According to the method reported earlier, the cells were fixed, plaques were counted, and virus titers were presented as plaque-forming units per milliliter (PFU/mL) (51).

### Time of addition experiment.

After infecting the cells with CHIKV (MOI 5), MBZM-N-IBT (100 μM) was added to the RAW 264.7 cells at 0, 1, 2, 3, 4, 5, 6, and 7 hpi. Ribavirin (10 μM) was used as a control. The supernatants were collected at 8 hpi, and viral titer was determined by plaque assay as mentioned before (21).

### Experiments before, during, and after treatment.

The RAW 264.7 cells were pretreated with MBZM-N-IBT (100 μM) for 2 h. Then the cells were washed properly with 1× PBS followed by infection. In case of during, the virus inoculum and the compound (100 μM) were incubated for 30 min and immediately used for infecting the cells for 1.5 h. After that, the cells were washed properly, and fresh medium was added. For post-treatment, after infection, the cells were washed and the compound was added. MBZM-N-IBT was present before and during infection for pre + during conditions. It was present before, during, and postinfection for pre + during + post conditions. Finally, the supernatants were collected at 8 hpi and subjected to plaque assay (58).

### Confocal microscopy.

The RAW 264.7 cells were seeded on coverslips in 6-well plates. The cells were infected after reaching 60% confluence and processed the confocal microscopy as described earlier (57). In brief, the cells were fixed at 8 hpi. CHIKV-E2 (1:750) was the primary antibody, and anti-mouse Alexa Flour 594 (1:1,000) was the secondary antibody (Invitrogen, MA). The cells were stained with 4′,6-diamidino-2-phenylindole (DAPI) (Life Technologies, Inc., CA) and mounted with antifade reagent (Invitrogen, MA). The images were acquired using the Leica confocal microscope (Leica Microsystems, Germany) with 20× objective and analyzed by Leica application suite (LASX) software.

### Expression and purification of CHIKV nsP2-CT.

The expression and purification of CHIKV-nsP2-CT was according to the previously described procedure (54) with some modifications. In brief, the transformed Rosetta 2 (DE3) cells were grown in Luria Bertani broth (HiMedia, India). The overexpression was induced with 0.4 mM isopropyl-d-1-thiogalactopyranoside (Sigma, MA) and allowed to proceed for 16 h at 18°C. The cell pellets after overexpression were collected by centrifugation and resuspended in lysis buffer having 50 mM Tris (pH 7.5), 500 mM NaCl, and 10 mM imidazole. The cells were disrupted by sonication using a Vibra-Cell probe-type ultrasonic processor (Sonics, CT). The lysed sample was clarified by centrifugation. The supernatant containing the soluble protein was passed through a pre-equilibrated HisTrap FF 5-mL nickel affinity column (Cytiva, MA). The column wash step was performed using a buffer containing 50 mM Tris (pH 7.5), 250 mM NaCl, 10 mM imidazole, and 1 mM phenylmethylsulfonyl fluoride (PMSF), and the bound protein was eluted with gradient against a buffer containing 50 mM Tris (pH 7.5), 250 mM NaCl, 250 mM imidazole, and 1 mM PMSF. The protein was then subjected to size exclusion chromatography using a HiLoad 16/600 Superdex 200-pg column (Cytiva) in a buffer containing 25 mM Tris (pH 7.5), 100 mM NaCl, 5%, glycerol, and 0.5 mM DTT. The peak fraction was analyzed by SDS-PAGE.

### MBZM-N-IBT and CHIKV nsP2-CT interaction study by fluorescence assay.

The interaction between the drug and protein has been studied by fluorimetric technique, which has been discussed previously (59). At a static temperature (25°C), different concentrations of the compound were titrated against a constant concentration of protein using a fluorimeter (Photon Technology International, NJ). An emission scan was performed from 310 to 370 nm against an excitation wavelength of 290 nm at slit width of 2 nm. 1 μM nsP2-CT was taken in a quartz cuvette and titrated against MBZM-N-IBT (1 to 100 μM), and subsequently the tryptophan fluorescence intensity was measured. A control was also subjected to the experiment using buffer and dimethyl sulfoxide (DMSO). The reading of fluorescence intensity at 335 nm was taken into consideration to calculate the *K_d_* value using a double reciprocal plot.

### CHIKV-nsP2-CT protease assay in the presence of MBZM-N-IBT.

The protease assay was performed in 96-well, black, flat-bottomed microtiter plates (Corning, NY) with a final volume of 100 μL. CHIKV-nsP2-CT at a final concentration of 1.5 μM was preincubated for 40 min at room temperature (RT) with different concentrations of the compounds in the assay buffer (20 mM Bis-Tris-propane, pH 7.5). The FRET substrate DABCYL-RAGG*YIFS-EDANS (GL Biochem, Shanghai, China) was added at a varying range of substrate concentrations (μM) to determine the protease kinetics. For determination of IC_50_, a specific substrate concentration (μM) was used. The enzymatic reaction mixture was incubated for 30 min at RT. The fluorescence intensity (excitation/emission, 355 nm/460 nm) of released EDANS was measured using a fluorometer as fluorescence units per unit of time (ΔRFU/s) (VICTOR Nivo; Perkin-Elmer, MA). The curve between RFU with the various substrate concentrations was plotted. The slope was determined to obtain FEC of given the peptide substrate. ΔRFU/s were normalized to the amount of the cleaved substrate per unit of time (μM/s) by FEC. *K_i_* was measured with increasing substrate concentrations (1, 10, 20, 50, 100, and 400 μM) and with a range of inhibitor concentrations (5, 10, and 50 μM) in a reaction mixture containing 1 μM CHIKV-nsP2-CT at 37°C for 30 min. The *K_i_* value was computed using GraphPad Prism 8.0 software by nonlinear regression of competitive enzyme kinetics. To determine the IC_50_ value of drug, the results were plotted as dose inhibition curves using nonlinear regression with a variable slope (with GraphPad Prism 8.0). For negative control, a fluoregenic peptide (DABCYL-GAEEWSLAIE-EDANS) that does not have the nsP2-CT cleavage site was also used (23). Telmisartan was used as a positive control as an nsP2 protease inhibitor (24).

### Animal studies.

The animal experiments were conducted under the strict guidelines defined by the Committee for the Purpose of Control and Supervision of Experiments on Animals (CPCSEA) of India. The Institutional Animal Ethics Committee (76/Go/Rebi/S/1999/CPCSEA, 28.02.17) reviewed and approved all procedures and experiments. The 10- to 12-day-old C57BL/6 mice were bred and housed under specific-pathogen-free conditions at the Animal House facility of the institute. For CHIKV infection, 10- to 12-day-old mice were infected with 1 × 10^6^ PFU of CHIKV-PS subcutaneously at the flank region of the right hind limb. PBS was injected in the same region of the mock mice. Based on an oral acute toxicity study in rats, the compound was categorized as an unclassified chemical (OECD-423 guidelines). This class of chemicals had an anticipated LD50 of 5,000 mg/kg. Thus, 1/100th of this (50 mg/kg) was considered safe for study in rats. Based on higher surface area to body weight, a dose of 100 mg/kg was considered safe for oral use in mice (60). Accordingly 2 h postinfection, different doses (5, 10, 15, 25, and 50 mg/kg) were given. This was continued at 24-h intervals up to 4 day postinfection (dpi), whereas the mock and infection-control group (*n* = 3 or 6) received only solvent. The reduction in viral titer was dose dependent up to 15 mg/kg. An increase in dose beyond this level failed to reduce viral titer further. After that, 15 mg/kg MBZM-N-IBT was used for all experiments. All the animals were monitored for disease symptoms every day. On 5 dpi, the mice were sacrificed, and serum was isolated from the blood. Different tissues were also collected and stored in 10% formalin for the histological experiment, in RNAlater (Invitrogen, MA, US) for RNA isolation, and snap-frozen in liquid nitrogen for Western blot analysis. To quantitate the viral titer, an equal quantity of tissue from each group was taken, and a homogeneous mixture was prepared in serum-free media (DMEM). After that, the mixture was subjected to syringe filtration using a 0.22-μm membrane and used for plaque assay. For the survival curve and clinical score studies, the above-mentioned infection and treatment protocols were followed (*n* = 6 mice in three groups). However, drug was administered up to 8 dpi. For this study, all mice were monitored every day up to 8 dpi. According to the symptom-based disease outcomes (no symptoms, 0; fur rise, 1; hunchback, 2; one hind limb paralysis, 3; both hind limb paralysis, 4; and death, 5), the clinical scores of each mice were tabulated on daily basis for the survival curve analysis (56, 58, 61).

### qRT-PCR.

The RAW 264.7 cells were seeded and infected with CHIKV as described above. The cells were harvested at 8 hpi to extract total RNA from the infected and treated samples. On the other hand, CHIKV-infected and treated mouse tissue of different organs stored as mentioned previously was used for RNA isolation. After that, total RNA extraction was performed using the TRIzol reagent (Invitrogen, MA) from the harvested cells and the tissues. The cDNA synthesis was carried out with an equal amount of RNA by using the First Strand cDNA synthesis kit (Invitrogen, MA), and qRT-PCR was performed using specific primers for the CHIKV-E1 and nsP2 genes (58). Glyceraldehyde 3-phosphate dehydrogenase (GAPDH) was taken as an endogenous control. Equal volume serum samples were collected for viral RNA extraction using the QIAamp viral RNA mini kit (Qiagen, Hilden, Germany) according to the manufacturer’s instructions. An equal volume of RNA was used to synthesize cDNA, and the E1 gene was amplified by qRT-PCR with equal volume of cDNA. The *C_t_* values were plotted against the standard curve to obtain CHIKV copy number (57).

### Western blot.

The Western blot analysis was performed to examine protein expression. In brief, virus-infected and drug-treated RAW 264.7 cells were harvested at 8 hpi. An equal volume of radioimmunoprecipitation assay (RIPA) buffer was used to lyse the cells. Snap-frozen mice tissue samples were homogenized by hand homogenizer and lysed in RIPA buffer using the syringe lysis method. An equal concentration of protein was separated on 10% SDS-polyacrylamide gel and was transferred onto a polyvinylidene difluoride (PVDF) membrane (Millipore, MA). Next, the membrane was probed with anti-nsP2 (1:1,000), anti-E2 (1:2,500) mAbs. All major MAPKs (p38 pAb, p-p38 pAb, JNK mAb, p-JNK mAb, ERK1/2 mAb, p-ERK1/2 mAb), p-cJUN mAb, p-ATF2/7 mAb, p-NF-κB mAb, NF-κB mAb, and COX-2 mAb were used in 1:1,000 dilution to probe the membrane. GAPDH mAb (1:3,000) and actin pAb (1:500) antibodies were used as loading controls. The blots were developed by the Immobilon Western chemiluminiscent horseradish peroxidase (HRP) substrate (Millipore, MA) in the ChemiDoc MP Imaging System (Bio-Rad Laboratories, CA), and the intensities of all of the protein bands were quantified from three independent experiments using ImageJ software (25, 58).

### Histopathological examinations.

The histopathological examinations were performed according to a previously described method (56, 58). In brief, 5-μm tissue sections were stained with hematoxylin and eosin (H&E) and visualized under a light microscope (Zeiss Vert. A1, Germany). Each section was also examined for the presence of CHIKV-E2 protein using specific antibody. The Alexa Fluor 594 (anti-mouse) (Invitrogen, MA) secondary Ab was also used for this. Then, the mounting reagent with DAPI (Invitrogen, MA) was applied to the slides and observed under the Apotome fluorescence microscope (Zeiss, Jena, Germany) (56, 58).

### Measurement of cytokine levels.

The mock, CHIKV-infected and drug-treated RAW 264.7 cell supernatants were collected at 8 hpi for this assay. In addition to that, every 1 to 5 dpi, serum samples from CHIKV-infected and drug-treated mice were collected. To measure the levels of cytokines (IL-12 [p40], MCP-1, IL-1β, IL-6, IP-10, KC, RANTES, GM-CSF, G-CSF, MIP-1β, and TNF-α), the magnetic bead-based Milliplex kit (MCYTMAG-70K-PX32) (Millipore, MA) was used according to the manufacturer’s instructions. The plate was read on the Luminex 200 plate reader with xPONENT software (Luminex Corporation, Austin, TX) (58).

### Proteome profiling.

To investigate the levels of different cytokines in mock, CHIKV-infected, and compound-treated mouse samples, proteome profiling was performed using a mouse XL cytokine array kit (R & D systems) as per the manufacturer’s instructions as described earlier (56). The array blots were incubated with serum samples and developed using the chemiluminescent HRP substrate and scanned by ChemiDoc MP imaging system (Bio-Rad Laboratories, CA). The relative intensity of the selected cytokines were quantified by ImageJ software (56).

### Isolation of human peripheral blood mononuclear cells (hPBMCs).

Isolation of human peripheral blood mononuclear cells (hPBMCs) was performed according to the procedure described earlier. Briefly, 30 mL blood was collected from each of three healthy individuals in sodium heparin Vacutainers (BD, NJ). Hi-Sep LSM (HiMedia, India) was used to isolate PBMCs from the blood samples. Isolated PBMCs were cultured in RPMI 1640 (PAN Biotech) supplemented with 15% FBS (PAN Biotech), l-glutamine (HiMedia, India), and antibiotic-antimycotic solution for 5 days. Every 2 days, the cells were washed and supplemented with fresh medium (58).

### Chikungunya virus infection in hPBMC and flow cytometric staining.

At the beginning, immunophenotyping was performed using fluorochrome-conjugated antibodies (anti-human CD3, CD11b, CD14, and CD19; procured from Abgenex India Pvt. Ltd., Bhubaneswar, India) by flow cytometry. After that, cytotoxicity assay of MBZM-N-IBT in adherent hPBMCs was carried out with the increasing concentrations of the compound by MTT assay as mentioned earlier. Then, 0.15 × 10^6^ cells were seeded in a 24-well plate. After 1 day, adherent cells were subjected to CHIKV infection at 5 MOI for 2 h, and the compound was added. At 12 hpi, infected and treated cells, as well as supernatants, were collected. The cells were fixed with 4% paraformaldehyde (PFA) and subjected to intracellular staining to detect viral protein E2. Supernatants were used to determine viral titer by plaque assay (58).

### Statistical analysis.

The one-way analysis of variance (ANOVA) and Dunnett’s multiple-comparison test was performed for all the experiments (*in vitro*, *in vivo*, and *ex vivo*). GraphPad Prism version 8.0.1 software was used to perform all the statistical analyses, in which *P* values are means ± SE (*n* = 3 or 6).
