# Supplementary material for: MBZM-N-IBT, a Novel Small Molecule, Restricts Chikungunya Virus Infection by Targeting nsP2 Protease Activity In Vitro, In Vivo, and Ex Vivo
Source: Antimicrob Agents Chemother. 2022 Jun 29;66(7):e00463-22. doi: 10.1128/aac.00463-22 (PMC9295557; doi:10.1128/aac.00463-22)
Supplement: Supplemental file 1 — Fig. S1 to S7 and Table S1. Download aac.00463-22-s0001.pdf, PDF file, 1.9 MB [file aac.00463-22-s0001.pdf]

MBZM-N-IBT a novel small molecule restricts Chikungunya virus infection by targeting nsP2 protease activity *in vitro, in vivo and ex vivo*

Saikat De[1, 2], Soumyajit Ghosh[1, 2 #], Supriya Suman Keshry[1, 3#], Chandan Mahish[4,5], Chinmayee Mohapatra[1] , Ankeeta Guru[1, 2], Prabhudutta Mamidi[1], Ankita Datey[1, 3], Sweta Smita Pani[1], Dileep Vasudevan[1], Tushar Kant Beuria[1], Subhasis Chattopadhyay[4], Bharat Bhusan Subudhi[6]* and Soma Chattopadhyay[1]*

[1] Institute of Life Sciences, Bhubaneswar, India

[2] Regional Centre for Biotechnology, Faridabad, India

[3] School of Biotechnology, Kalinga Institute of Industrial Technology (KIIT) University, Bhubaneswar, India.

[4] National Institute of Science Education and Research, Bhubaneswar 752050, Odisha, India

[5] Homi Bhabha National Institute, Training School Complex, Anushaktinagar, Mumbai 400094, India

[6] School of Pharmaceutical Sciences, Siksha O Anusandhan Deemed to be University, Bhubaneswar, India

[#] These authors contributed equally to this work (based on alphabetical order)

*Address of the Corresponding authors:

Bharat Bhusan Subudhi

School of Pharmaceutical Sciences, Siksha O Anusandhan Deemed to be University

Khandagiri Square, Bhubaneswar, Odisha-751030, India

Phone No: +91-9853945363; Email: bharatbhusans@gmail.com

And

Soma Chattopadhyay

Infectious Disease Biology, Institute of Life Sciences (Autonomous Institute of Department of Biotechnology, Government of India), Nalco Square, Bhubaneswar, Odisha-751023, India

Phone No: +91-674-2304235; Email: sochat.ils@gmail.com

Running Title: MBZM-N-IBT inhibits CHIKV infection and inflammation

Key Word: Chikungunya, Anti-viral, Infection, Inflammation, Replication

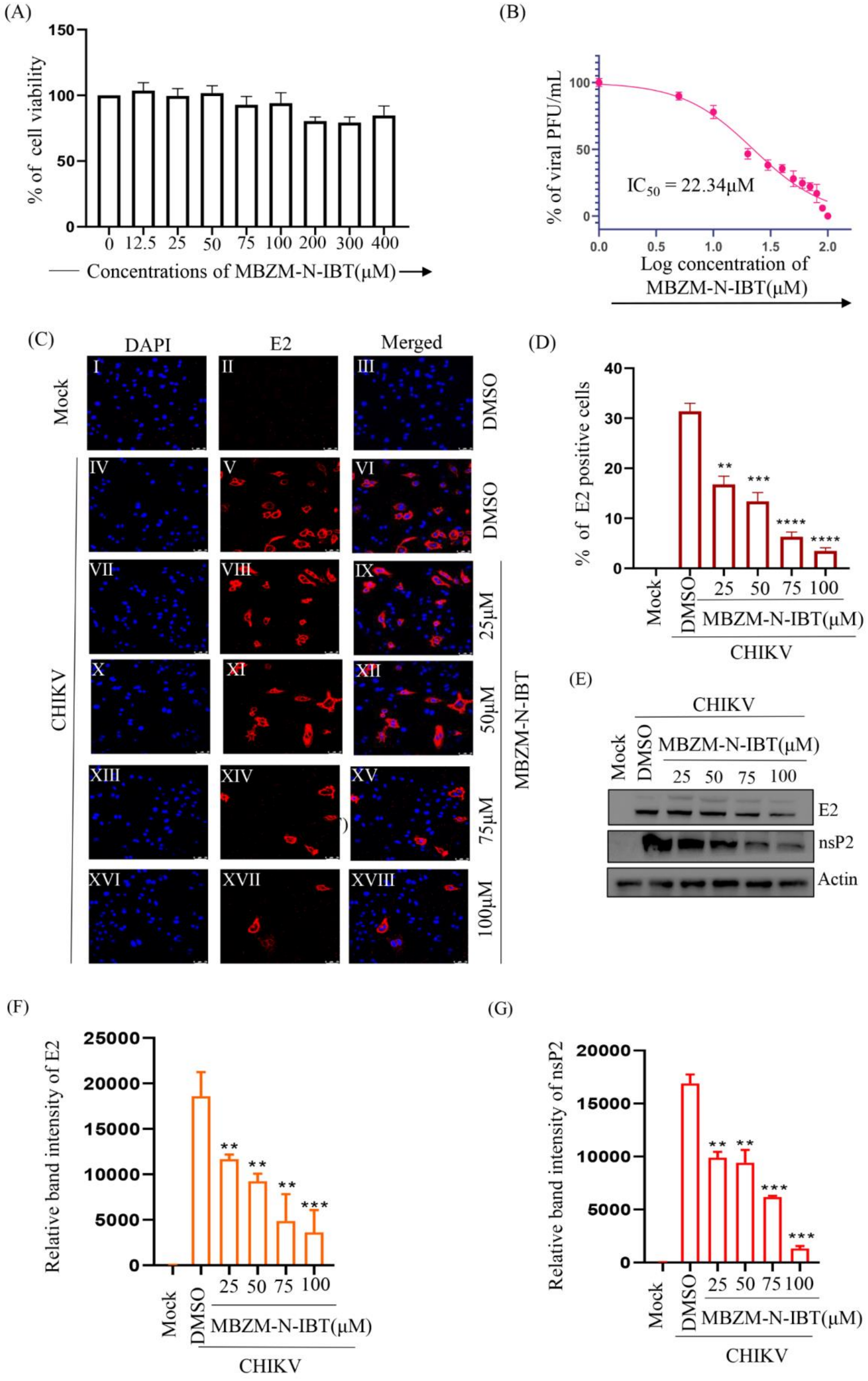
(A)
150
100
50
0
% of cell viability
0
12.5
25
50
75
100
200
300
400
Concentrations of MBZM-N-IBT(μM)
(B)
% of viral PFU/mL
100
50
0
0.0
0.5
1.0
1.5
2.0
IC50 = 22.34μM
Log concentration of
MBZM-N-IBT(μM)
(C)
DAPI
E2
Merged
Mock
CHIKV
DMSO
DMSO
25μM
50μM
75μM
100μM
MBZM-N-IBT
I
II
III
IV
V
VI
VII
VIII
IX
X
XI
XII
XIII
XIV
XV
XVI
XVII
XVIII
(D)
40
30
20
10
0
% of E2 positive cells
**
***
****
****
Mock
DMSO
25
50
75
100
MBZM-N-IBT(μM)
CHIKV
(E)
CHIKV
Mock
DMSO
MBZM-N-IBT(μM)
25
50
75
100
E2
nsP2
Actin
(F)
25000
20000
15000
10000
5000
0
Relative band intensity of E2
**
**
**
***
Mock
DMSO
25
50
75
100
MBZM-N-IBT(μM)
CHIKV
(G)
20000
15000
10000
5000
0
Relative band intensity of nsP2
**
**
***
***
Mock
DMSO
25
50
75
100
MBZM-N-IBT(μM)
CHIKV

Figure S1. MBZM-N-IBT inhibits CHIKV infection efficiently in mice monocyte/macrophage (RAW 264.7) cells. (A) Bar diagram showing the viability of RAW 264.7 cells in presence of different concentrations of MBZM-N-IBT. (B) RAW 264.7 cells were infected with CHIKV and different concentrations (10µM, 20µM, 30µM, 40µM, 50µM, 60µM, 70µM, 80µM, 90µM, 100µM) of compound was added. The supernatants were collected at 8 hpi and virus titers were determined by plaque assay. The line diagram represents the IC_{50} value of the compound in CHIKV infected RAW 264.7 cells where the X-axis depicts the logarithmic value of different concentrations of the compound and Y-axis depicts the percentage of PFU/mL. (C) RAW 264.7 cells plated onto the cover-slips were infected with CHIKV and compound was added with different concentrations (25µM, 50µM, 75µM, 100µM). After 8 hpi the cells were fixed and probed with E2 antibody followed by staining with secondary antibody, anti-mouse Alexa Fluor 594 (red). Nuclei were counterstained with DAPI (blue). (D) Bar diagram representing the percent positive E2 cell counts from the confocal images. (E) RAW 264.7 cells were infected with CHIKV, treated with different concentrations of compound (25µM, 50µM, 75µM, 100µM) and were harvested at 8 hpi. Cell lysates were processed for Western blot using antibodies against CHIKV nsP2 and E2 proteins. Actin was used as a loading control. (F, G) Bar diagrams showing the relative band intensities of CHIKV-E2 and CHIKV-nsP2 protein levels. Data represented as mean ± SEM (n=3, $*p \leq 0.05$ was considered statistically significant).

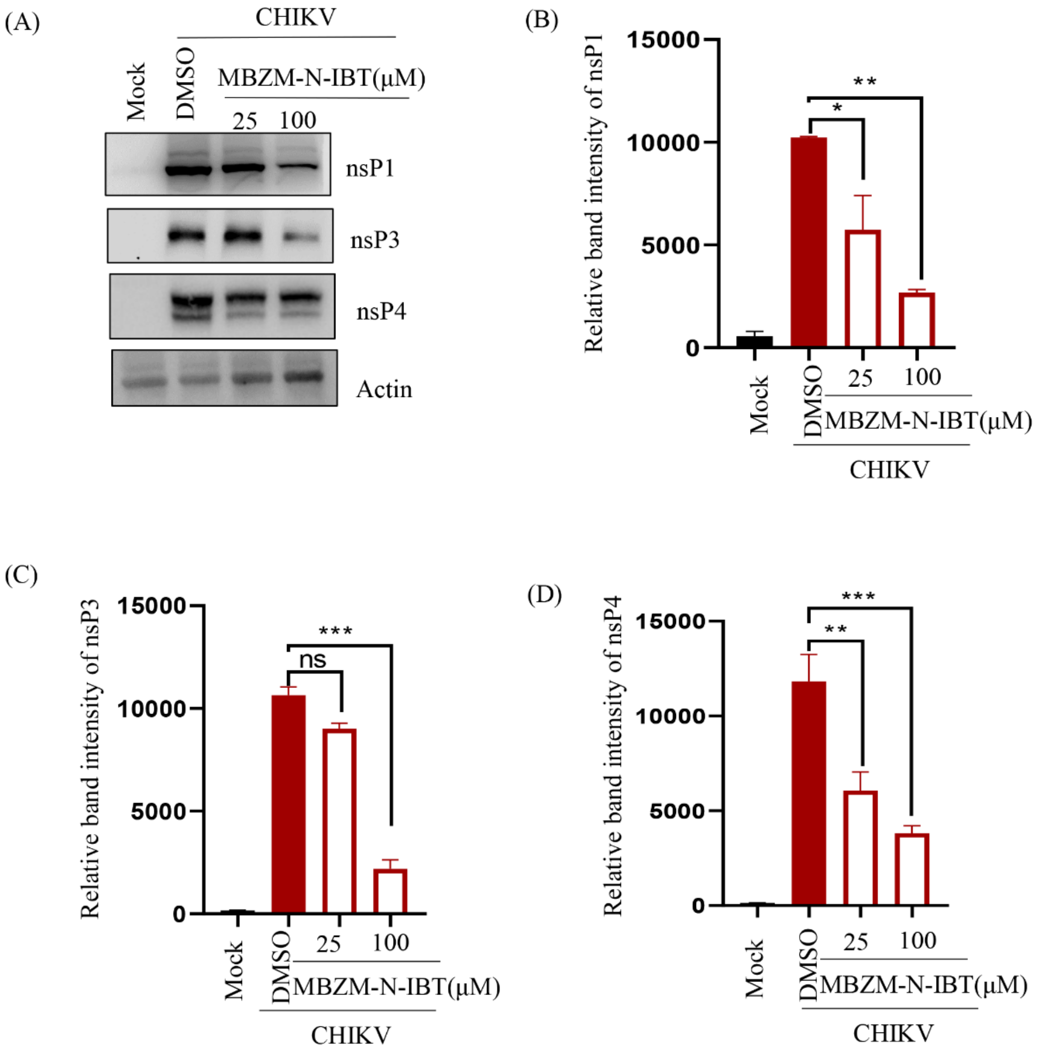

Figure S2. MBZM-N-IBT reduces all other non-structural proteins in CHIKV infected mice monocyte/macrophage (RAW 264.7) cells. RAW 264.7 cells were infected with CHIKV and different concentrations (25µM and 100µM) of compound was added. Mock, infected and treated cells were harvested at 8 hpi. (A) Cell lysates were processed for Western blot using antibodies against CHIKV nsP1, nsP3 and nsP4 proteins. Actin was used as a loading control. (B, C, D) Bar diagrams describing the relative band intensities of CHIKV-nsP1, CHIKV-nsP3 and CHIKV-nsP4 protein levels respectively. Data represented as mean ± SEM (n=3, *$p \leq 0.05$ was considered statistically significant).

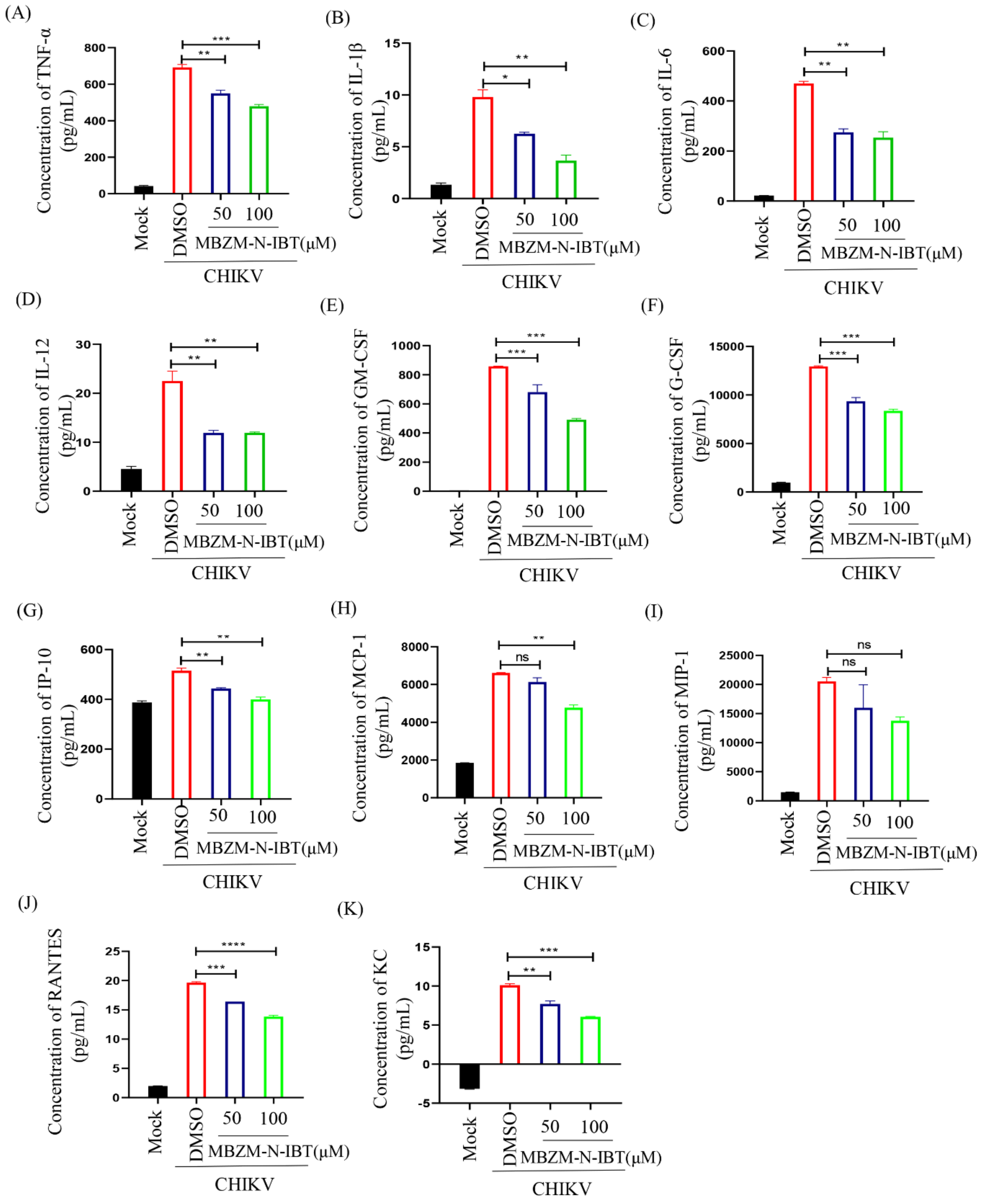

(A)
Concentration of TNF-α (pg/mL)
800
600
400
200
0
***
**
Mock
DMSO
50
100
MBZM-N-IBT(μM)
CHIKV
(B)
Concentration of IL-1β (pg/mL)
15
10
5
0
**
*
Mock
DMSO
50
100
MBZM-N-IBT(μM)
CHIKV
(C)
Concentration of IL-6 (pg/mL)
600
400
200
0
**
**
Mock
DMSO
50
100
MBZM-N-IBT(μM)
CHIKV
(D)
Concentration of IL-12 (pg/mL)
30
20
10
0
**
**
Mock
DMSO
50
100
MBZM-N-IBT(μM)
CHIKV
(E)
Concentration of GM-CSF (pg/mL)
1000
800
600
400
200
0
***
***
Mock
DMSO
50
100
MBZM-N-IBT(μM)
CHIKV
(F)
Concentration of G-CSF (pg/mL)
15000
10000
5000
0
***
***
Mock
DMSO
50
100
MBZM-N-IBT(μM)
CHIKV
(G)
Concentration of IP-10 (pg/mL)
600
400
200
0
**
**
Mock
DMSO
50
100
MBZM-N-IBT(μM)
CHIKV
(H)
Concentration of MCP-1 (pg/mL)
8000
6000
4000
2000
0
**
ns
Mock
DMSO
50
100
MBZM-N-IBT(μM)
CHIKV
(I)
Concentration of MIP-1 (pg/mL)
25000
20000
15000
10000
5000
0
ns
ns
Mock
DMSO
50
100
MBZM-N-IBT(μM)
CHIKV
(J)
Concentration of RANTES (pg/mL)
25
20
15
10
5
0
****
***
Mock
DMSO
50
100
MBZM-N-IBT(μM)
CHIKV
(K)
Concentration of KC (pg/mL)
15
10
5
0
-5
***
**
Mock
DMSO
50
100
MBZM-N-IBT(μM)
CHIKV

Figure S3. MBZM-N-IBT reduces the CHIKV induced pro-inflammatory cytokine production in RAW 264.7 cells. The RAW 264.7 cells were infected with CHIKV, treated with two different concentrations of MBZM-N-IBT (50µM and 100µM) and were harvested at 8 hpi. Cell supernatants were subjected to the Milliplex assay to quantitate the pro-inflammatory cytokine production. (A, B, C, D, E, F) Bar diagrams showing the levels of secreted cytokines (TNF-α, IL-1β, IL-6, IL-12, GM-CSF, G-CSF). (G, H, I, J, K) Bar diagrams indicating the levels of chemokines (IP10, MCP-1, MIP-1, RANTES, and KC). Data represented mean ± SEM (n=3). $*p \leq 0.05$ was considered as statistically significant.

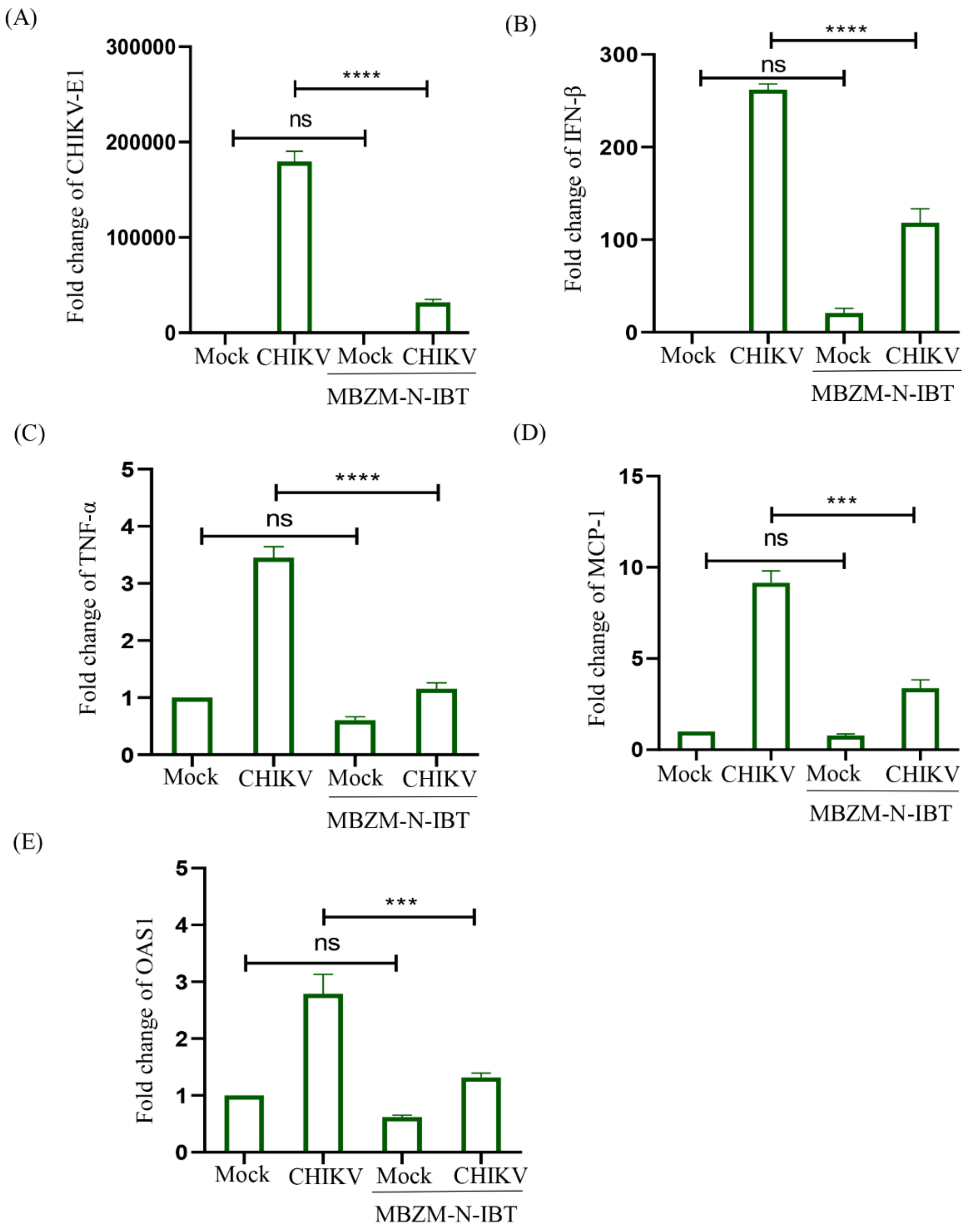

(A)
Fold change of CHIKV-E1
300000
200000
100000
0
ns
****
Mock CHIKV Mock CHIKV
MBZM-N-IBT
(B)
Fold change of IFN-β
300
200
100
0
ns
****
Mock CHIKV Mock CHIKV
MBZM-N-IBT
(C)
Fold change of TNF-α
5
4
3
2
1
0
ns
****
Mock CHIKV Mock CHIKV
MBZM-N-IBT
(D)
Fold change of MCP-1
15
10
5
0
ns
***
Mock CHIKV Mock CHIKV
MBZM-N-IBT
(E)
Fold change of OAS1
5
4
3
2
1
0
ns
***
Mock CHIKV Mock CHIKV
MBZM-N-IBT

Figure S4. MBZM-N-IBT has no effect on immunomodulatory or antiviral host factors. The RAW 264.7 cells infected with CHIKV (MOI-5) were treated with different concentrations of the compound and harvested at 8 hpi. Whole cell RNA was extracted by TRIzol® and qRT-PCR was performed. (A-E) Bar diagram representing the fold change of E1, IFN-β, TNF-α, MCP-1, OAS1 genes after CHIKV infection and drug treatment. Data represented as mean ± SEM (n=3, $p \leq 0.05$ was considered statistically significant).

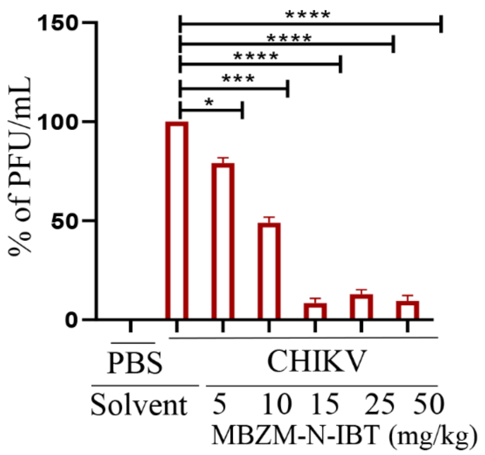

Figure S5. Calculation of optimum does of MBZM-N-IBT in mice. C57BL/6 mice were infected subcutaneously with 10^6 PFU of CHIKV and treated with increasing concentrations of the compound (5, 10, 15, 25, 50 mg/kg) at every 24h intervals upto 4 dpi. All mice were sacrificed at 5 dpi after that equal amount of muscle tissue from each group of mice were homogenized, filtered and subjected to plaque assay. Bar diagram showing the percentage of viral titer in CHIKV infected mice treated with increasing concentrations of compound. Data represented as mean ± SEM (n=3, $*p \leq 0.05$ was considered statistically significant).

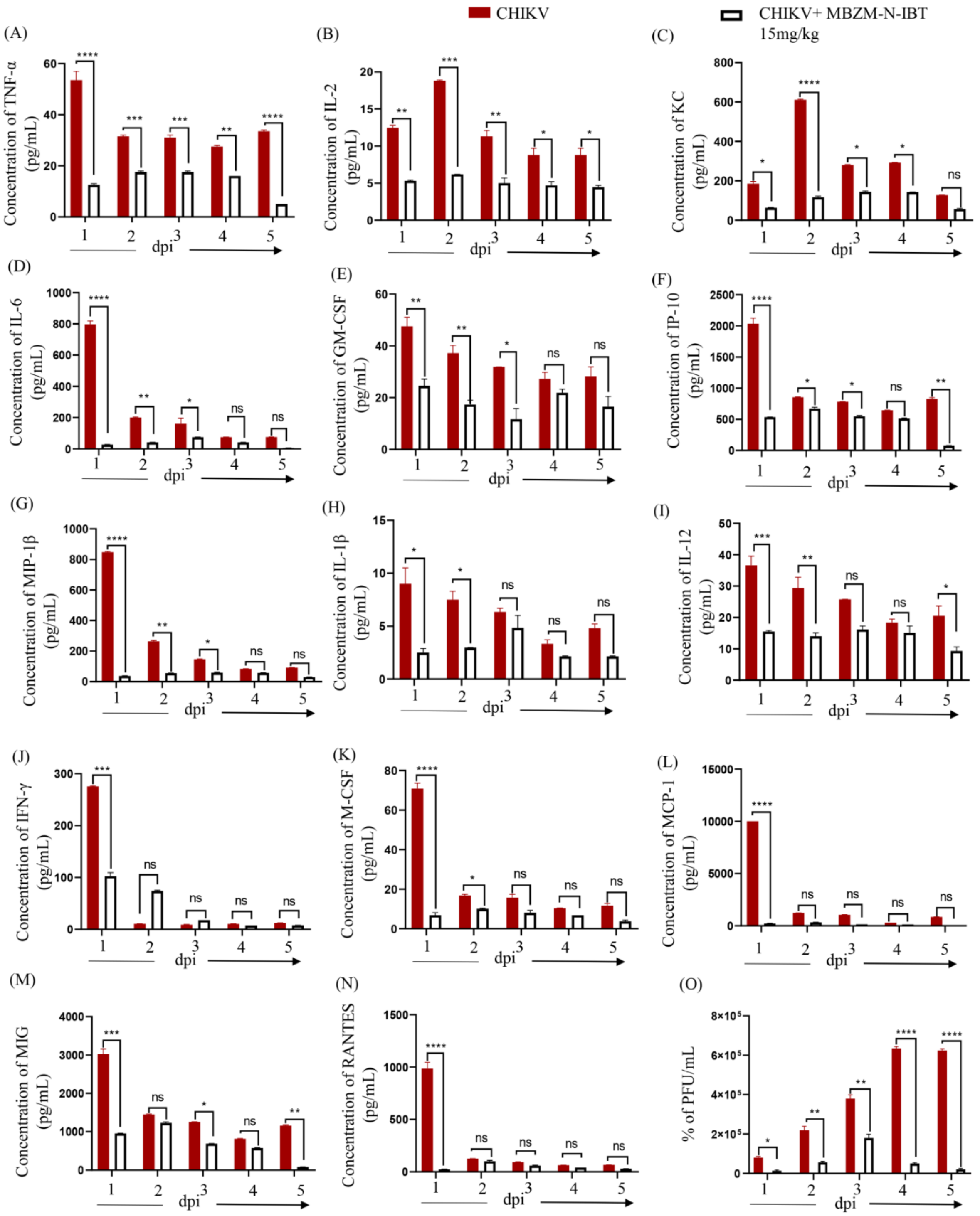
CHIKV
CHIKV+ MBZM-N-IBT 15mg/kg
(A)
Concentration of TNF-α (pg/mL)
(B)
Concentration of IL-2 (pg/mL)
(C)
Concentration of KC (pg/mL)
(D)
Concentration of IL-6 (pg/mL)
(E)
Concentration of GM-CSF (pg/mL)
(F)
Concentration of IP-10 (pg/mL)
(G)
Concentration of MIP-1β (pg/mL)
(H)
Concentration of IL-1β (pg/mL)
(I)
Concentration of IL-12 (pg/mL)
(J)
Concentration of IFN-γ (pg/mL)
(K)
Concentration of M-CSF (pg/mL)
(L)
Concentration of MCP-1 (pg/mL)
(M)
Concentration of MIG (pg/mL)
(N)
Concentration of RANTES (pg/mL)
(O)
% of PFU/mL
dpi

Figure S6. MBZM-N-IBT treatment dampens the induction of proinflammatory cytokines and chemokines that are upregulated by CHIKV infection in mice. C57BL/6 mice were infected subcutaneously with 10^6 PFU of CHIKV and treated with 15mg/kg MBZM-N-IBT at every 24h intervals up to 4 dpi. 3 mice were sacrificed at each dpi (1 dpi-5 dpi), serum was collected and subjected to Milliplex assay. (A, B, C, D, E, F G, H, I, J, K, L, M and N) Bar diagrams showing the levels of secreted cytokines and chemokines (TNF-α, IL-2, KC, IL-6, GM-CSF, IP-10, MIP-1β, IL-1β, IL-12, IFN-γ, M-CSF, MCP-1, MIG and RANTES). Data represented mean ± SEM (n=3). $*p \leq 0.05$ was considered as statistically significant. (O) Equal amount (weight) of hind limb muscle tissue from each group of mice were homogenized in serum free media (DMEM) after that it was filtered and subjected to plaque assay. Bar diagram showing the percentage of viral titer in CHIKV infected and treated mice. Data represented as mean ± SEM (n=3, $*p \leq 0.05$ was considered statistically significant).

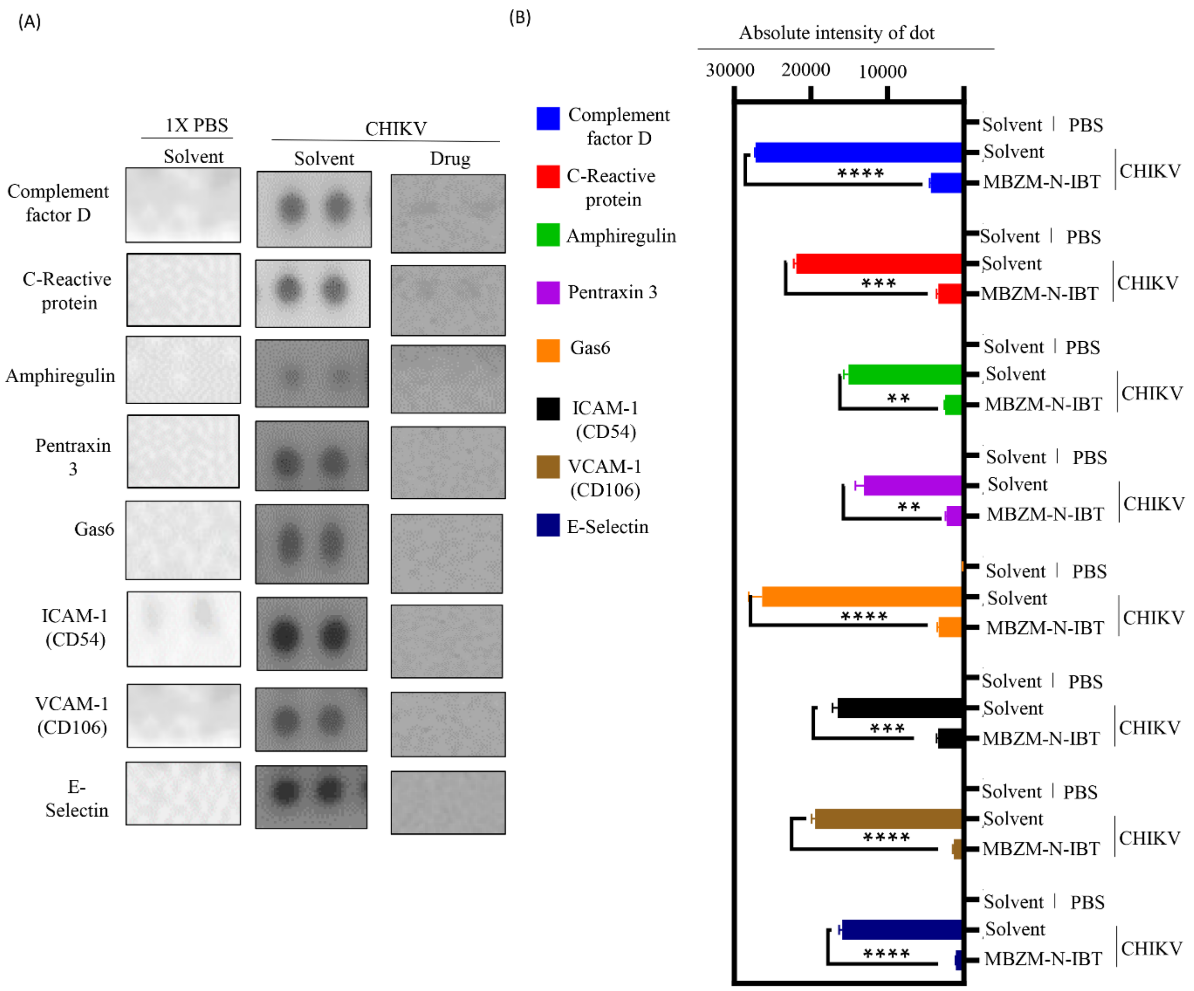

Figure S7. MBZM-N-IBT treatment downregulates the proinflammatory molecules that are upregulated by CHIKV infection in mice. C57BL/6 mice were infected subcutaneously with 10^6 PFU of CHIKV and treated with 15 mg/kg MBZM-N-IBT at every 24h intervals upto 5 dpi. Mice was sacrificed at 6 dpi. Serum was isolated from the blood of each group of mice (n=6) and subjected to proteome profiler assay. (A) Image panel showing the dot blot of different proinflammatory molecules. (B) Bar diagram showing the absolute dot intensity of each molecules. Data represented as mean ± SEM (n=3, *$p \leq 0.05$ was considered statistically significant).

Table S1. Primers name and sequences.

Sl no.	Gene Name	Primers name	Sequences
1.	CHIKV-E1	CL11 F	5′-TGCCGTCACAGTTAAGGACG-3′
		CL12 R	5′-CCTCGCATGACATGTCCG-3′
2.	IFN-β	IFNB F	5′CCTCTCCATCAACTATAAGC3′
		IFNB R	5′CAACAATAGTCTCATTCCAC3′
3.	TNF-α	TNFA F	5′GAGCACAGAAAGCATGATCC 3′
		TNFA R	5′CCATAGAACTGATGAGAGGG 3′
4.	MCP-1	MCP F	5′-CAGCCAGATGCAGTTAACGC-3′
		MCP R	5′-CAGACCTCTCTCTTGAGCTTGG-3′
5.	OAS1	OAS F	5′ GTGGAAAGAAGAGGTCCTG3′
		OAS R	5′GTATCAGTCTCAGGACAGC3′
6.	GAPDH	GAPDH F	5′-CAAGGTCATCCATGACAACTTTG-3′
		GAPDH R	5′-GTCCACCACCCTGTTGCTGTAG-3′
